# Using BLAST to Teach “E-value-tionary” Concepts

**DOI:** 10.1371/journal.pbio.1001014

**Published:** 2011-02-01

**Authors:** Cheryl A. Kerfeld, Kathleen M. Scott

**Affiliations:** 1 Joint Genome Institute, Walnut Creek, California, United States of America; 2 Department of Plant and Microbial Biology, University of California, Berkeley, California, United States of America; 3 Integrative Biology Department, University of South Florida, Tampa, Florida, United States of America; University of California Berkeley/JGI, United States of America

## Introduction

BLAST (Basic Local Alignment Search Tool; [1],[2]) is the key bioinformatic tool for sequence comparison and retrieval from databases. BLAST is often the first step in using sequence-based information to design experiments and contextualize experimental results. Its speed and ease of use help account for this, with experiments requiring simply submitting a sequence of interest (the query sequence) and waiting a few seconds. At the same time, by rendering thinking unnecessary, BLAST is often used suboptimally, with many experienced researchers simply using the default parameters because they do not know how to manipulate them or accepting results with little understanding of their full meaning (or lack thereof).

Recognition of the importance of BLAST to modern life sciences has led to its use in many biology courses, even at the high school level, to introduce students to bioinformatics applications in the life sciences. Concepts of molecular evolution (e.g., gene duplication and divergence; orthologs versus paralogs) are quite abstract and are best communicated with examples (Box 1). It is possible to use case studies from the literature, but the abundance of sequence data present in public databases raises the far more attractive possibility of using searches tailored to a particular course, or, better yet, allowing the students to choose their own examples.

**Box 1. Concepts at a Glance**

**Leads into biological sciences**

- Molecular evolution (e.g., homologs, paralogs, orthologs)
- Gene alignment
- Domain structure of proteins
- Biochemical nature of amino acids: “frequent” and “infrequent” substitutions
- Conserved versus divergent regions of genes

**Leads into math**

- Amino acid identity matrices
- Quantification of sequence identity and similarity

**Leads into information technology**

- Database queries and their tradeoffs (speed versus completeness)
- Large dataset management

Less obviously, another benefit of teaching students how the BLAST algorithm works is that it provides an opportunity to illustrate how mathematics functions as a language of biology. For example, given that BLAST has been designed to retrieve homologs, there are several steps in the BLAST program that incorporate molecular evolution concepts to maximize the possibility of finding sequences with a shared evolutionary history. More basically, understanding the steps in the calculation of an E-value provides an opportunity to show the relationship between how the algorithm works and fundamental principles of biochemistry and evolution. Here, we provide an approach to teaching the basics of BLAST to students in order to emphasize how the algorithm translates fundamental biological principles into numerical terms culminating in an E value. Acquiring a feel for the algorithm and exploring genomic sequence data has the potential to inform a student's grasp of biomedical, biochemical, and biogeochemical concepts, presenting an excellent opportunity for multidisciplinary integration.

## Explaining the Relationship between Aligning Sequences and Evolutionary Biology

**Figure 1:**
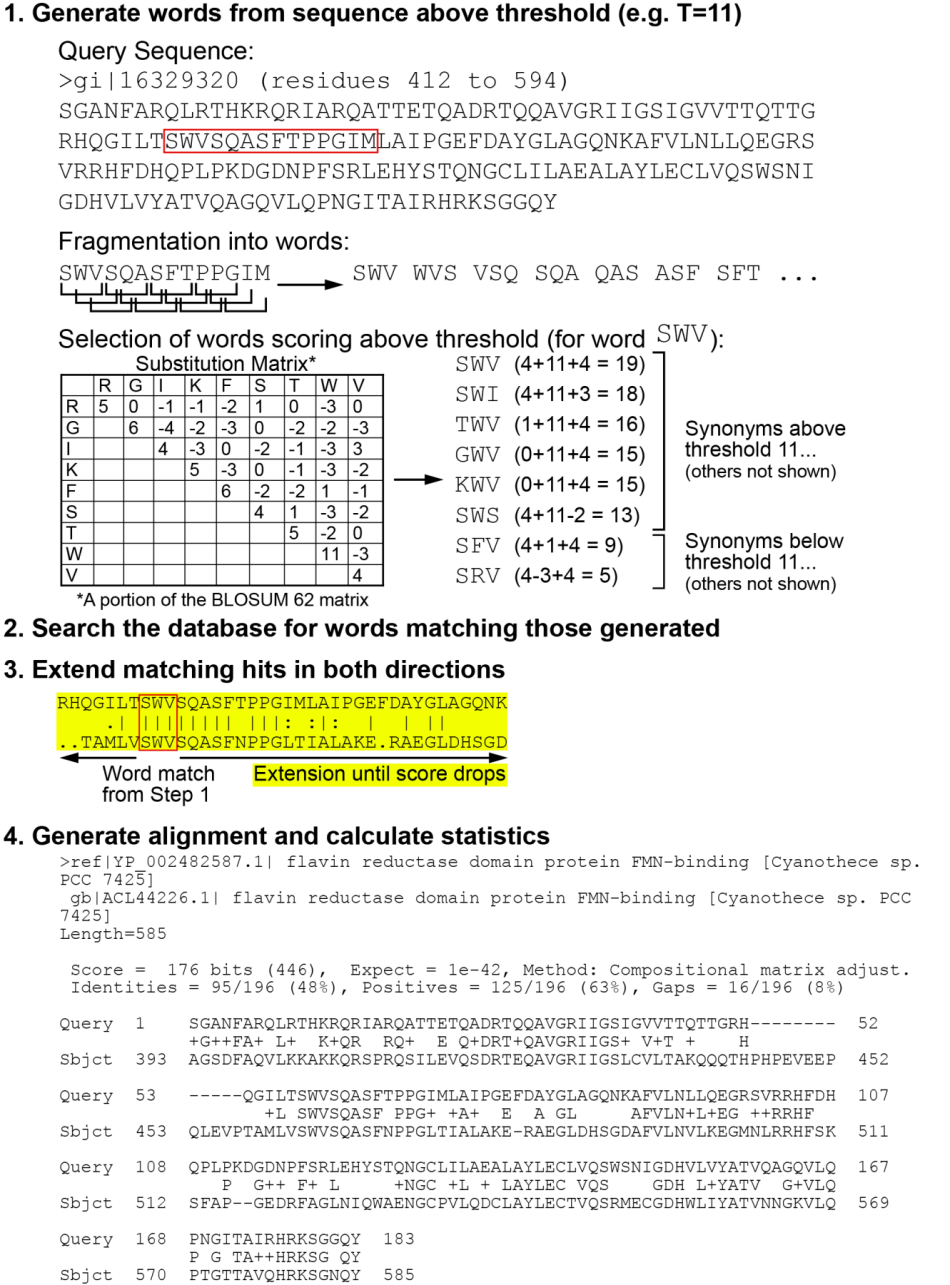
Steps in the BLAST algorithm.

To begin a BLAST search of a database, the user provides a query sequence, which is a nucleotide (nt) or amino acid (aa) sequence for which they are interested in finding homologs. The BLAST algorithm begins by fragmenting the sequence into “words” (e.g., from the National Center for Biotechnology Information (NCBI) interface, 16–256 nt, or 2–3 aa), and, from each word, creating a set of acceptable “synonyms” that represent possible changes in sequence due to mutation (Figure 1). Words and their synonyms are scored with respect to how well they match the query sequence, based on substitution matrices (see below) from curated alignments of gene/protein families (e.g., BLOcks of Amino Acid SUbstitution Matrix [BLOSUM]; [3],[4]). The words that match sufficiently well to have a score above a set threshold value are carried forward to compare to all the sequences in the database being searched for homologs. All sequences in the database are then scanned for the presence of these words; sequences carrying two matches within a preset distance from each other (which suggests a conserved region shared by both query and subject sequences) are set aside until the entire database has been scanned. This “short list” of subject sequences is then carried forward by extending the alignment outward from the words to determine whether the match between the query and subject sequences extends beyond the local match between the subject sequence and the word. Initial “rough” alignments are extended without gaps to verify that the sequences match beyond the word hits. If the threshold score for the “ungapped” alignment is high enough that it suggests that the two sequences are indeed homologs, a second alignment is undertaken in which gaps (see below) are allowed to optimize the alignment. The sequences retrieved after these steps are referred to as the “subject” sequences.

The extent of the sequence similarity between the subject and query sequences is reported as a raw score, *S*. A pairwise alignment between a subject sequence and query sequence in which the two have a high degree of sequence similarity will have a high *S* value. *S* is calculated from the alignment of the two sequences by adding the scores from each pair *ij* of residues in the alignment: 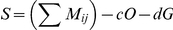
in which *M* is the score from a similarity matrix (e.g., BLOSUM62; more on this below) for a particular pair of amino acids, *c* is the number of gaps, *O* is the penalty for the existence of a gap, *d* is the total length of the gaps, and *G* is the per-residue penalty for extending the gap.

Gaps are spaces introduced into either the query or subject sequence to optimize alignments. In terms of the underlying biology of the two aligned sequences, gaps correspond to regions in which there is an insertion or a deletion in one of the amino acid sequences relative to the other. In a protein BLAST, the default setting for *O* is 11, while for *G* it is 1. It is useful to ask students to consider why the gap existence penalty is larger than the gap extension penalty. The answer is that it is assumed that that a gap corresponds to either an insertion or deletion event, regardless of the size of the gap. Hence, a larger penalty is imposed for the existence of the gap whereas the length of that insertion or deletion is relatively less significant. Furthermore, these insertion and deletion events are likely rare and so more heavily penalized. If they were not, and the gap opening penalty correspondingly lowered, numerous gaps would be included in alignments; taken to the extreme, this would allow any two sequences to be aligned.

Alignments are extended position by position with concomitant scoring until the match falls below a threshold score, at which point it is terminated. It is important to note that alignment between the query and subject sequences does not have to cover the full length of the two sequences. Therefore, retrieved subject sequences commonly align with only a portion of the query sequence—it is this “local” rather than global quality that is more than nominally BLAST's strength. Underscoring this distinction for students provides another opportunity to discuss protein evolution. In considering their results, they may need to evaluate if they have found homology in one domain of a multidomain protein. BLAST provides a list of potential homologs to the query sequence that is based on regions of sequence similarity. Matches that occur over limited regions of these sequences provide an illustration of both the modular nature of protein structure (domains), as well as the modular nature of protein evolution (new domain combinations). Protein domains with a common ancestry are shared among many protein families whose members have divergent functions. For example, flavin reductase domains occur in the many enzymes that transfer electrons from a reduced compound (e.g., NADH) to FMN or FAD. If two amino acid sequences have a flavin reductase domain but do not share other domains with common ancestry, the two sequences will align with a high score at the flavin reductase domain, but will not align meaningfully or significantly elsewhere (Figure 1).

Sometimes it is helpful to “mask” parts of the query sequence to prevent them from being aligned with subject sequences. Masking is helpful when the query sequence has “low-complexity” regions, such as stretches of small hydrophobic amino acids that are commonly present in transmembrane helices of integral membrane proteins. Because these features arose from convergent evolution, and their inclusion in BLAST searches could result in spurious hits, it is best to set the BLAST search parameters to eliminate these sorts of regions from word generation, as well as alignment scoring.

## Why Aren't Raw Scores Enough? The Calculation of the E-Value

Because one has the option of using different parameters (e.g., matrices) in different BLAST experiments, it is ideal to report results in such a way as to be able to compare alignments made with different scoring matrices or gap penalties. To do this, *S*' values (bit scores) are calculated:
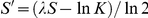
in which *λ* and *K* reflect the matrices and penalties used. If analyses were to stop here, one would have a list of sequences sorted by bit scores that would reflect the degree of similarity to the query sequence. The question of whether the sequences were homologs to the query sequence would not yet be directly addressed: how high does a bit score have to be to suggest shared ancestry? Larger databases are more likely to include sequences with matches to the query that are due to chance, not homology.

To address this issue, E-values are calculated from bit scores as 
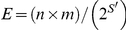
in which *n* is the total number of residues (amino or nucleic acids) in the database, and *m* is the length of the query sequence. E-values are the number of subject sequences that can be expected to be retrieved from the database that have a bit score equal to or greater than the one calculated from the alignment of the query and subject sequence, based on chance alone. E-values for subject sequences that are very similar to the query sequence will be quite small, and are widely used as a means to assess the confidence with which one should claim the subject sequence(s) and the query sequence as homologs. Collectively, the parameters for a BLAST search are given at the bottom of the output; going through these with students provides another opportunity to reinforce these concepts and explain why an E-value for BLAST searches using the same protein sequence will change over time.

## Substitution Matrices and Protein Biochemistry

Substitution matrices are critical at two steps in the BLAST process: 1) in the evaluation of “words” with which to tag subject sequences for further scrutiny, and 2) in the extension and evaluation of alignments between the query sequence and a subject sequence. These matrices are used to calculate scores for alignments, which are used to gauge the strength of the match between two sequences. Scores are calculated by comparing residues at each position in the alignment. This series of scores is summed over the length of the alignment and used to calculate the raw score.

It is intuitive that having an exact match in residues at many positions in an alignment should result in a high alignment score; it is less obvious how these matches should be weighted among different residues (is a leucine–leucine match as informative as a tryptophan–tryptophan match?), as well as how severely residue substitutions should be penalized (should a leucine–isoleucine substitution be penalized to the same degree as a leucine–tryptophan substitution?).

Substitution matrices were created to resolve these “gray areas”; they consist of factors to use to weigh residue matches and substitutions. BLOSUM matrices are commonly used to compare amino acid sequences and were constructed from curated alignments of amino acid sequences [4]. The alignments were scanned for positions at which sequences diverged, and the frequencies of substitutions for each amino acid were noted; some amino acid substitutions were frequently observed (e.g., due to chemical similarity between residues) and therefore should be penalized less stringently than those rarely observed. Amino acid “commonness” was also considered; exact matches between more pedestrian amino acids (e.g., leucine) are not scored as highly as more “exotic” ones (e.g., tryptophan).

Different levels of overall sequence divergence also affect amino acid substitution frequencies (e.g., fewer substitutions are expected for less divergent sequences; therefore, the substitutions that do occur should be penalized more heavily). To account for this effect, BLOSUM matrices were constructed from alignments with different degrees of divergence. BLOSUM62 was created from an alignment of sequences with at least 62% sequence identity, while BLOSUM80 used an alignment with 80% [4].

Examining and comparing substitution matrices with students provides an opportunity to consider not only the consequences of residue substitution but also the reason(s) for the different substitution penalties, allowing them to apply what they know about amino acid chemistry. When comparing two BLOSUM matrices, students will need to consider the types of amino acid replacements expected to accumulate between recently versus deeply diverging sequences.

## Meaning and Results

Students (and researchers as well) tend to draw an arbitrary line below which they consider E-values to provide convincing evidence that two sequences are homologs (e.g., E<0.00001). It is informative to scrutinize this assumption, and ask the students to consider whether and when more stringent E-values might be appropriate (e.g., to assist in sorting paralogs from orthologs), or when larger E-values do not provide definitive evidence of evolutionary independence (as is the case when two sequences share an ancient ancestor).

It is also informative for the students to discuss what their alignments “mean”, and whether the pairwise alignments between their query sequence and the subject sequences “prove” whether the sequences are homologs. Indeed, it can catalyze a larger discussion of whether it is possible to “prove” that two sequences are homologs, and what other approaches (e.g., protein structure, gene context) might be used to strengthen or refute such an assertion.

A meaningful alignment will facilitate the comparison of two sequences with a shared evolutionary history by maximizing the juxtaposition of similar and identical residues. Sequences with a recent shared ancestry will have a high degree of similarity; their alignments will have many identical residues, few substitutions and gaps, and tiny E-values. Conversely, sequences with an ancient common ancestor will be deeply divergent, with few shared sequence identities, many gaps, and larger E-values. Furthermore, an alignment of two sequences can clarify which portions are conserved (e.g., active sites), and which are divergent, which helps cultivate students' understanding of protein structure and function.

In summary, deconstructing the BLAST algorithm and manipulating parameters systematically and evaluating the results with students helps them understand not only what the scores mean but also how to manipulate parameters to optimize their searches. There is a wealth of additional resources available (Box 2; Supporting Information S1 and Text S1). Finally, explicating the algorithm in this way allows students to explore research databases thoughtfully and illustrates the critical connection between mathematics and science, showing how numbers can be used to quantify biological relationships from the level of gene to organism (Box 3). As bioinformatics and databases will increasingly become part of the undergraduate life sciences curriculum, new opportunities are emerging to teach biological principles through their bioinformatic articulations.

**Box 2. Teaching Tools**

- Powerpoint slides for teaching BLAST (Supporting Information S1)

**Online resources**

- National Center for Biotechnology Information (NCBI): http://www.ncbi.nlm.nih.gov/guide/
  - BLAST interface to query vast protein and nucleotide sequence databases, and protein structure databases
  - Links to PubMed
  - Tutorials for resources
- Bioinformatics tutorials at the European Bioinformatics Institute (EBI): http://www.ebi.ac.uk/2can/tutorials/index.html
  - BLAST background http://www.ebi.ac.uk/2can/tutorials/protein/blast.html
- Integrated Microbial Genomes (IMG): http://img.jgi.doe.gov/cgi-bin/pub/main.cgi
  - BLAST interface to query sequenced microbial genomes
  - Simple tools for genome: genome comparison
- PFAM: http://pfam.sanger.ac.uk/
  - BLAST interface to query curated protein families
  - Comprehensive background information and literature about protein families

**Box 3. Evaluation Tools**

**Questions to ask before the lesson**

- What is a homolog?
- How can you tell if two genes are likely to share a common ancestor?
- How can you tell if two proteins share a common domain?
- Is the degree of harm of a mutation in a gene sensitive to its position in a gene?

**Questions to ask after the lesson**

- Can you ever prove that two genes share a common ancestor? Why or why not?
- How could you build a strong argument that two genes have the same or similar function in two different organisms?
- Can you, in your own words, describe the process of a BLAST search?
- Which steps in a BLAST search help to make it happen so quickly?

## Supporting Information

Supporting Information S1 **Powerpoint file--Using BLAST To Teach “E-value-tionary” Concepts.**
(PPT)

Text S1 **Acquiring a feel for the algorithm: manipulating the parameters of BLAST and other experiments.**
(DOCX)

## Abbreviations

aa: amino acid
BLAST: Basic Local Alignment Search Tool
NCBI: National Center for Biotechnology Information
nt: nucleotide
