# Supplementary material for: Using BLAST to Teach “E-value-tionary” Concepts
Source: PLoS Biol. 2011 Feb 1;9(2):e1001014. doi: 10.1371/journal.pbio.1001014 (PMC3032543; doi:10.1371/journal.pbio.1001014)
Supplement: Supporting Information S1 — Powerpoint file--Using BLAST To Teach “E-value-tionary” Concepts. (PPT) [file pbio.1001014.s001.ppt]

## Slide 1
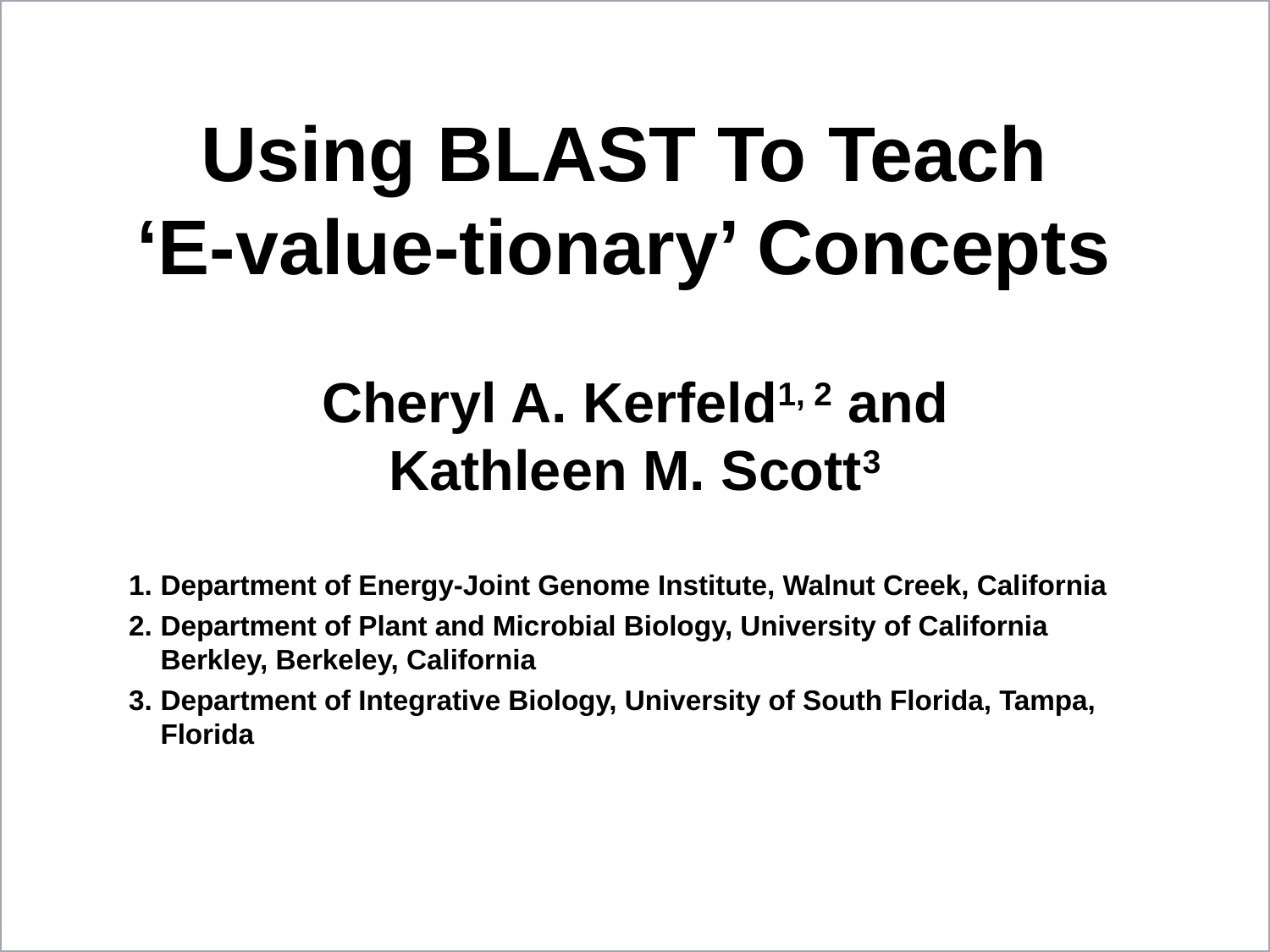

# Using BLAST To Teach ‘E-value-tionary’ Concepts
Cheryl A. Kerfeld1, 2 and Kathleen M. Scott3
Department of Energy-Joint Genome Institute, Walnut Creek, California
Department of Plant and Microbial Biology, University of California Berkley, Berkeley, California
Department of Integrative Biology, University of South Florida, Tampa, Florida

## Slide 2
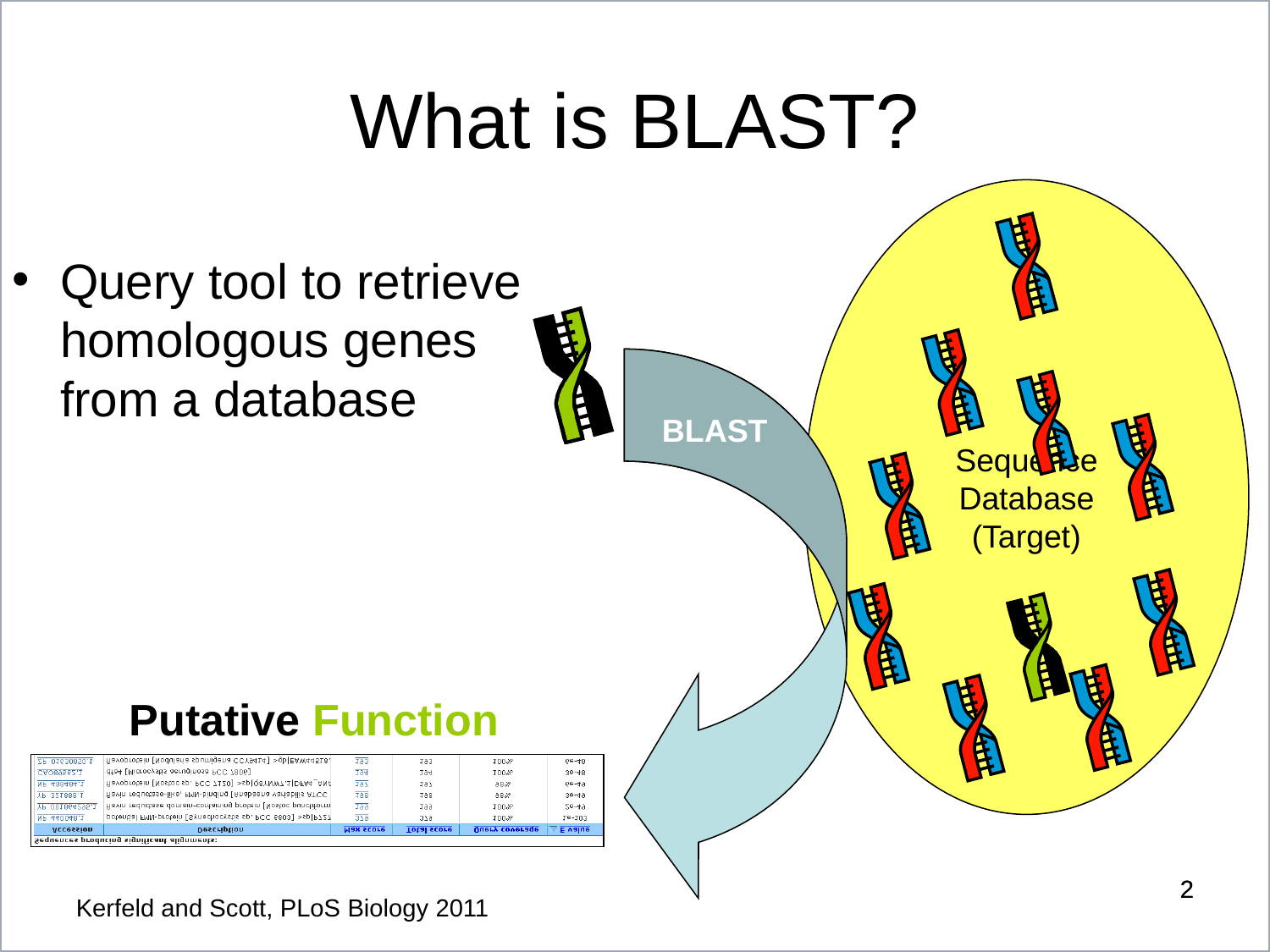

# What is BLAST?
Sequence
Database
(Target)
Query tool to retrieve homologous genes from a database
BLAST
Putative Function
<number>
<number>
Kerfeld and Scott, PLoS Biology 2011

## Slide 3
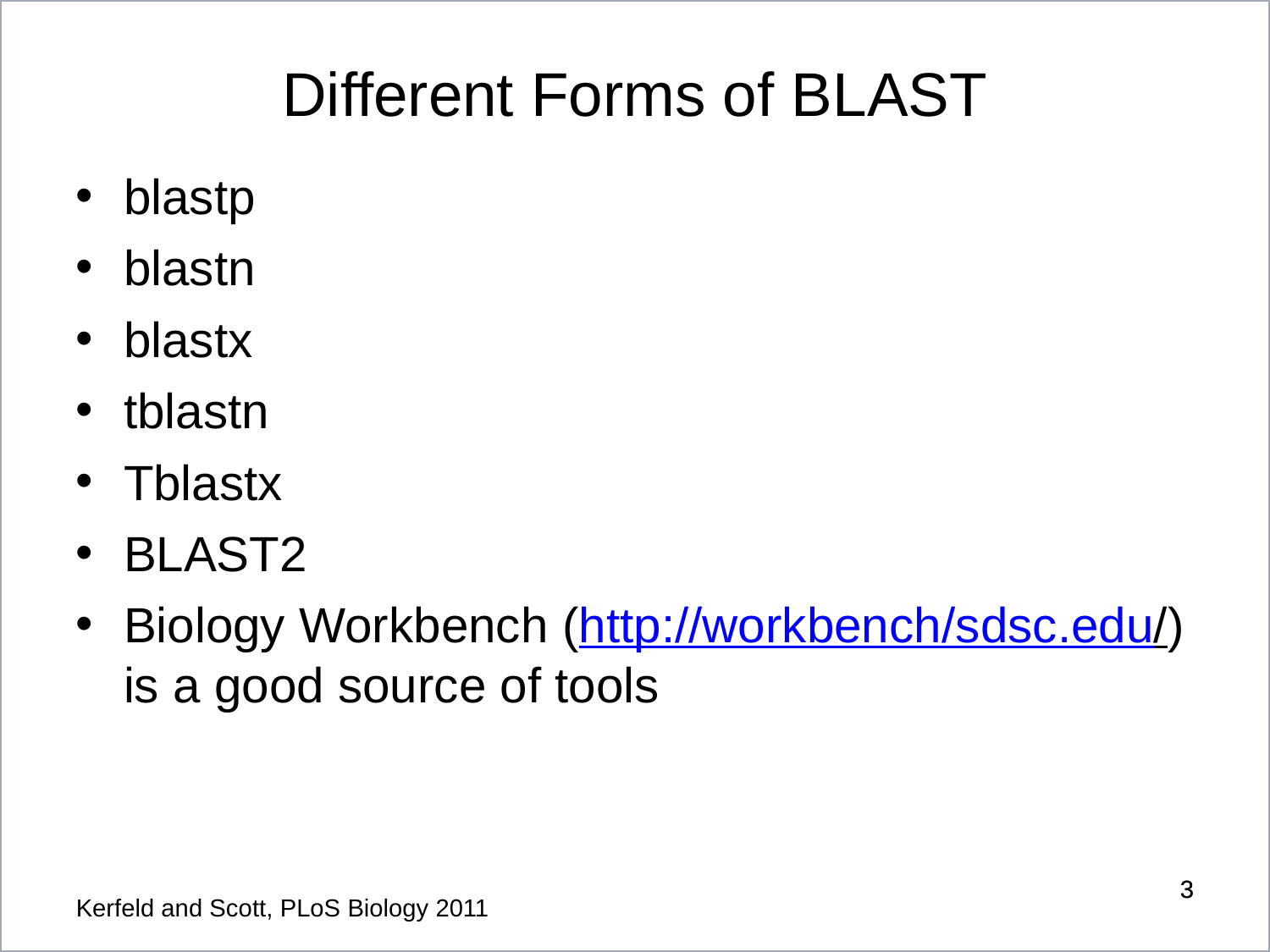

# Different Forms of BLAST
blastp
blastn
blastx
tblastn
Tblastx
BLAST2
Biology Workbench (http://workbench/sdsc.edu/) is a good source of tools
<number>
<number>
Kerfeld and Scott, PLoS Biology 2011

## Slide 4
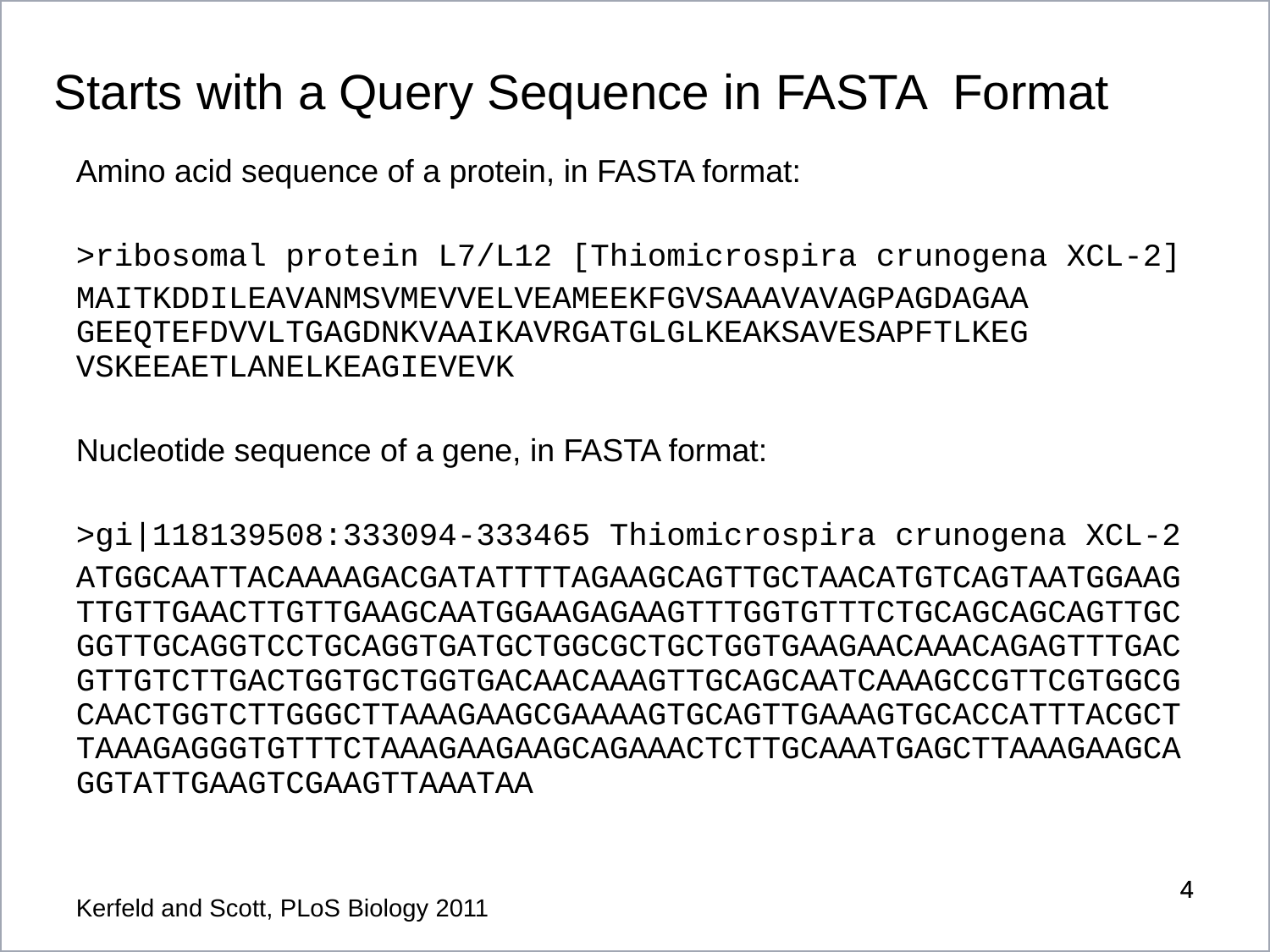

# Starts with a Query Sequence in FASTA Format
Amino acid sequence of a protein, in FASTA format:
>ribosomal protein L7/L12 [Thiomicrospira crunogena XCL-2]
MAITKDDILEAVANMSVMEVVELVEAMEEKFGVSAAAVAVAGPAGDAGAA GEEQTEFDVVLTGAGDNKVAAIKAVRGATGLGLKEAKSAVESAPFTLKEG VSKEEAETLANELKEAGIEVEVK
Nucleotide sequence of a gene, in FASTA format:
>gi|118139508:333094-333465 Thiomicrospira crunogena XCL-2
ATGGCAATTACAAAAGACGATATTTTAGAAGCAGTTGCTAACATGTCAGTAATGGAAGTTGTTGAACTTGTTGAAGCAATGGAAGAGAAGTTTGGTGTTTCTGCAGCAGCAGTTGCGGTTGCAGGTCCTGCAGGTGATGCTGGCGCTGCTGGTGAAGAACAAACAGAGTTTGACGTTGTCTTGACTGGTGCTGGTGACAACAAAGTTGCAGCAATCAAAGCCGTTCGTGGCGCAACTGGTCTTGGGCTTAAAGAAGCGAAAAGTGCAGTTGAAAGTGCACCATTTACGCTTAAAGAGGGTGTTTCTAAAGAAGAAGCAGAAACTCTTGCAAATGAGCTTAAAGAAGCAGGTATTGAAGTCGAAGTTAAATAA
<number>
<number>
Kerfeld and Scott, PLoS Biology 2011

## Slide 5
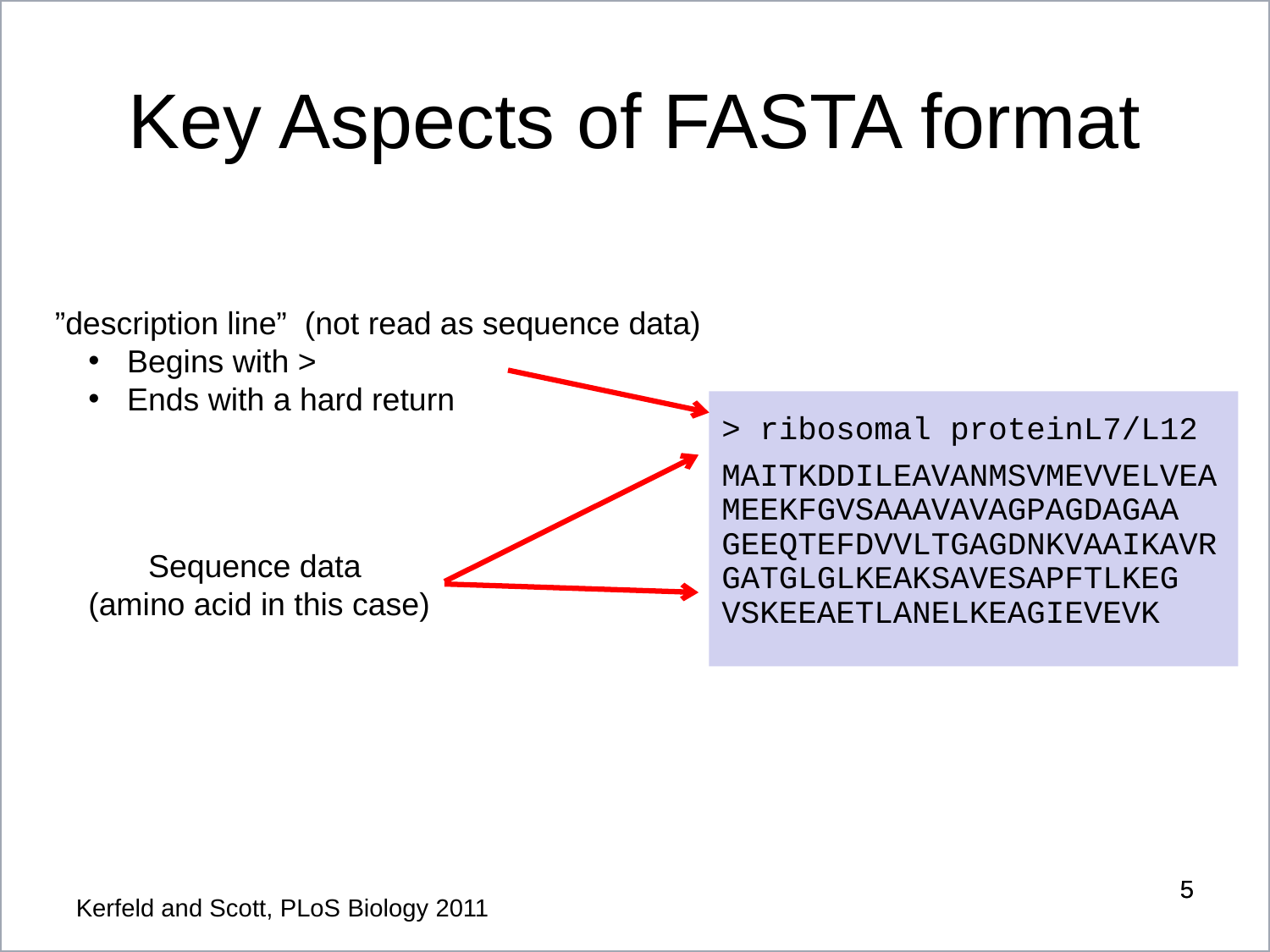

# Key Aspects of FASTA format
”description line” (not read as sequence data)
 Begins with >
 Ends with a hard return
> ribosomal proteinL7/L12
MAITKDDILEAVANMSVMEVVELVEAMEEKFGVSAAAVAVAGPAGDAGAA GEEQTEFDVVLTGAGDNKVAAIKAVRGATGLGLKEAKSAVESAPFTLKEG VSKEEAETLANELKEAGIEVEVK
Sequence data
(amino acid in this case)
<number>
<number>
Kerfeld and Scott, PLoS Biology 2011

## Slide 6
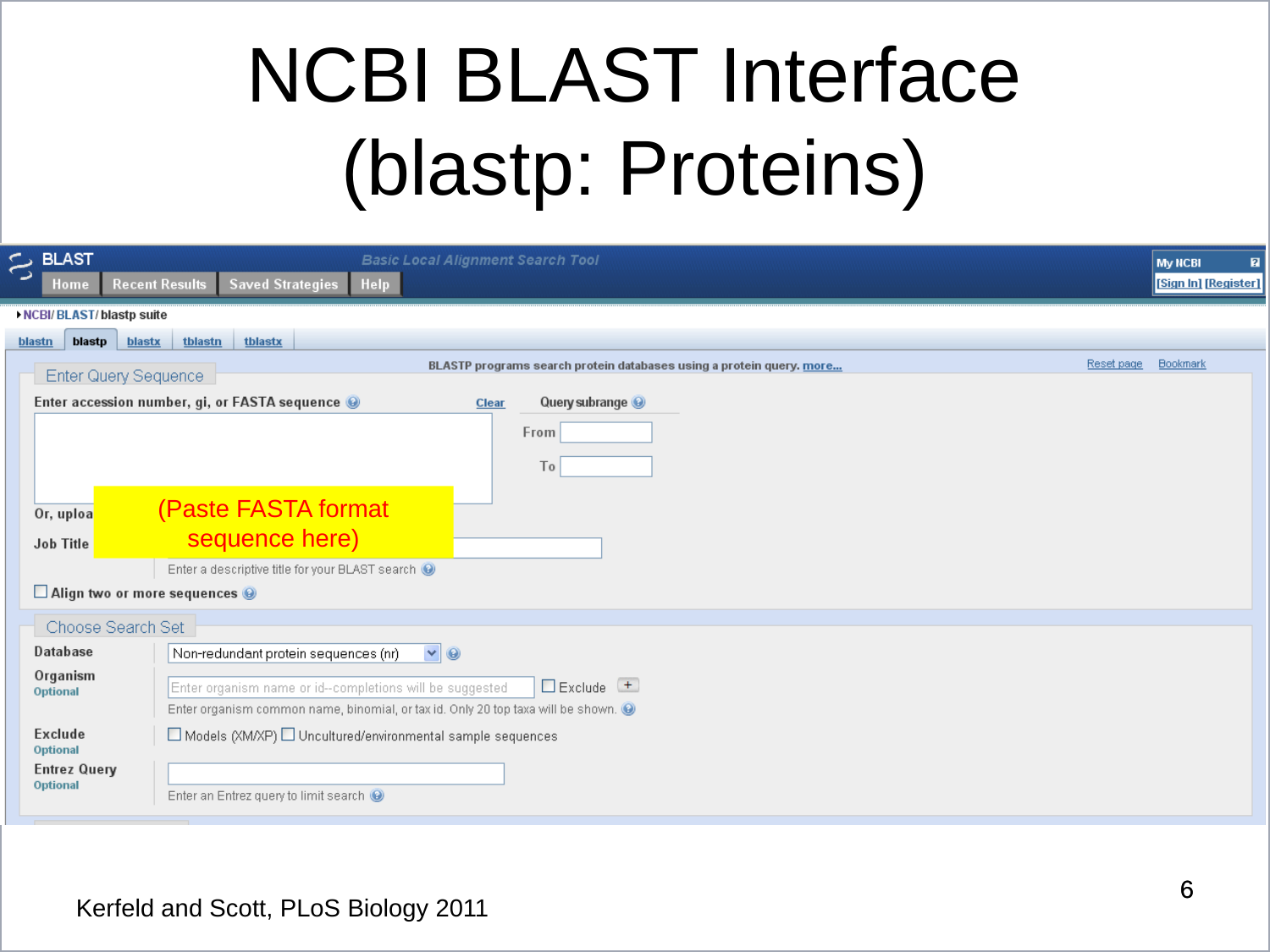

# NCBI BLAST Interface (blastp: Proteins)
(Paste FASTA format
sequence here)
<number>
<number>
Kerfeld and Scott, PLoS Biology 2011

## Slide 7
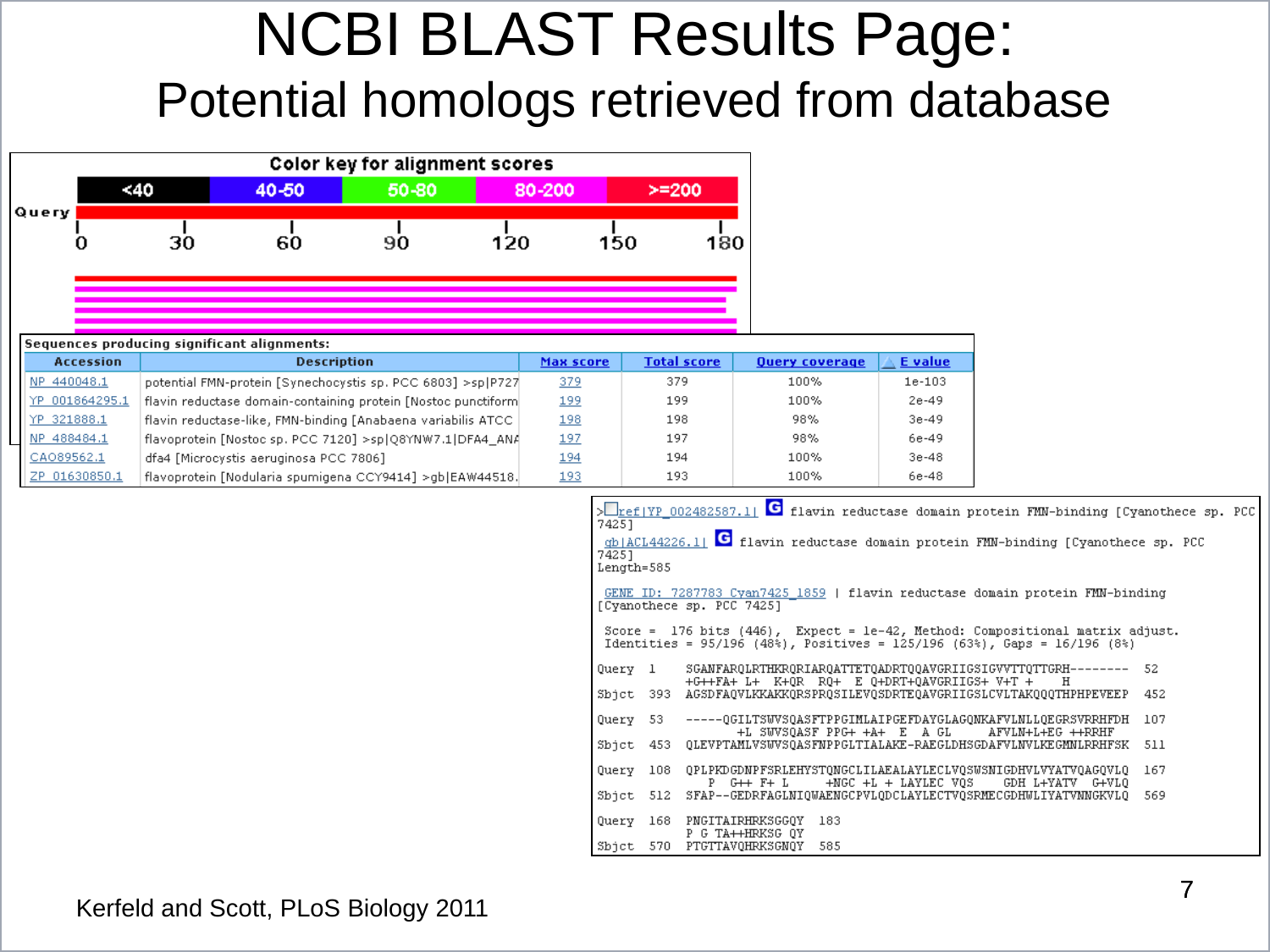

# NCBI BLAST Results Page: Potential homologs retrieved from database
<number>
<number>
Kerfeld and Scott, PLoS Biology 2011

## Slide 8
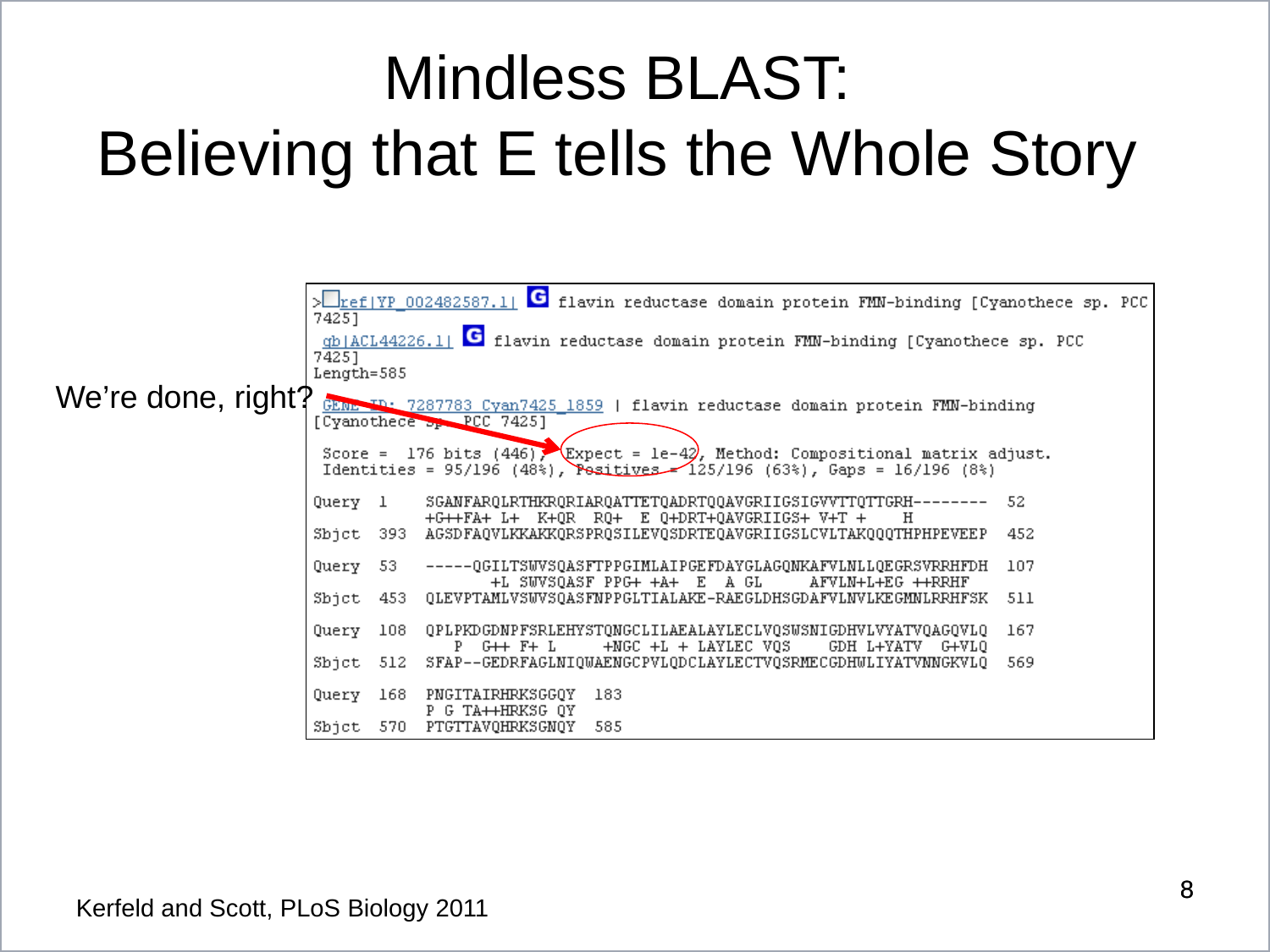

# Mindless BLAST: Believing that E tells the Whole Story
We’re done, right?
<number>
<number>
Kerfeld and Scott, PLoS Biology 2011

## Slide 9
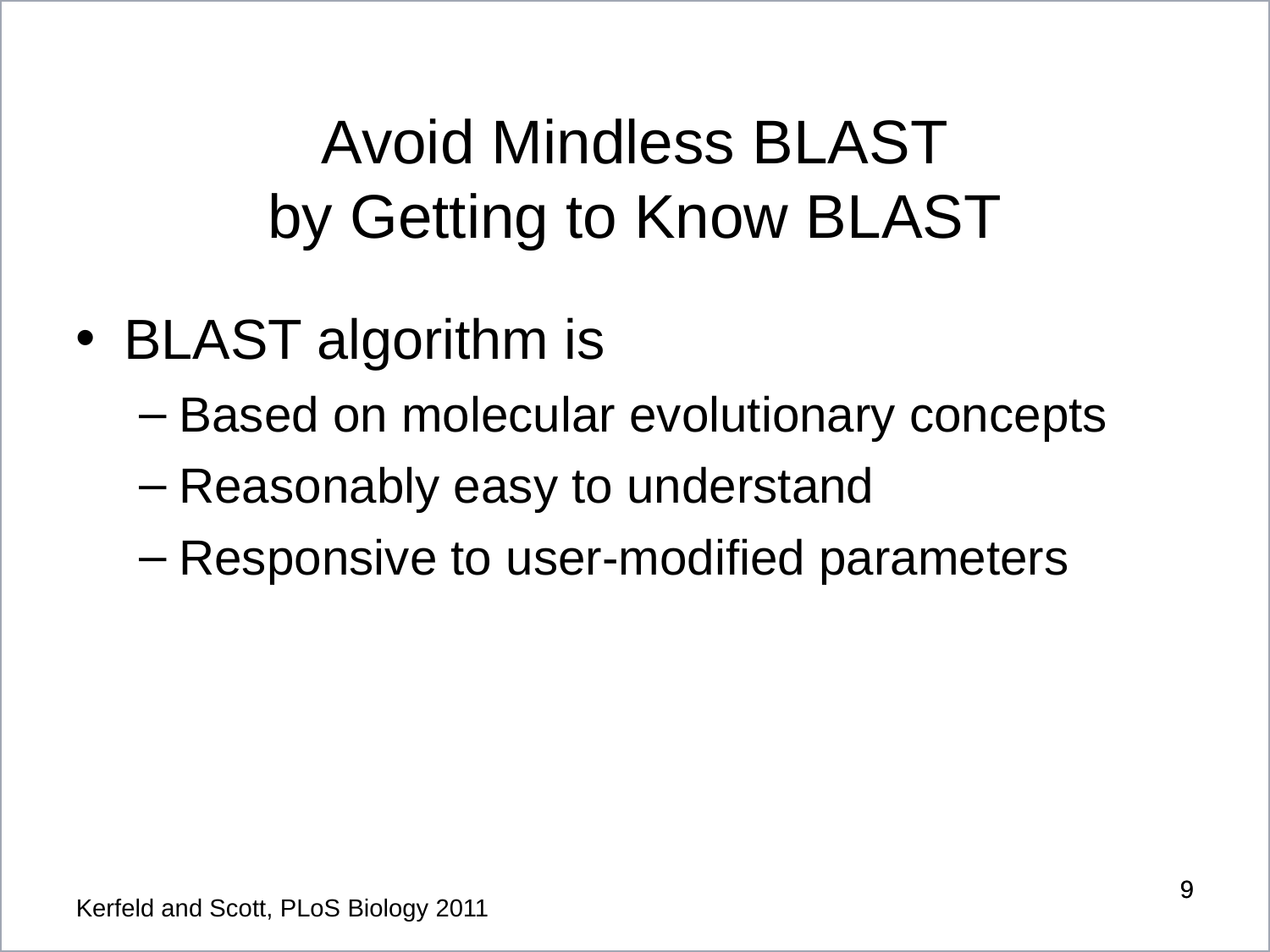

# Avoid Mindless BLAST by Getting to Know BLAST
BLAST algorithm is
Based on molecular evolutionary concepts
Reasonably easy to understand
Responsive to user-modified parameters
<number>
<number>
Kerfeld and Scott, PLoS Biology 2011

## Slide 10
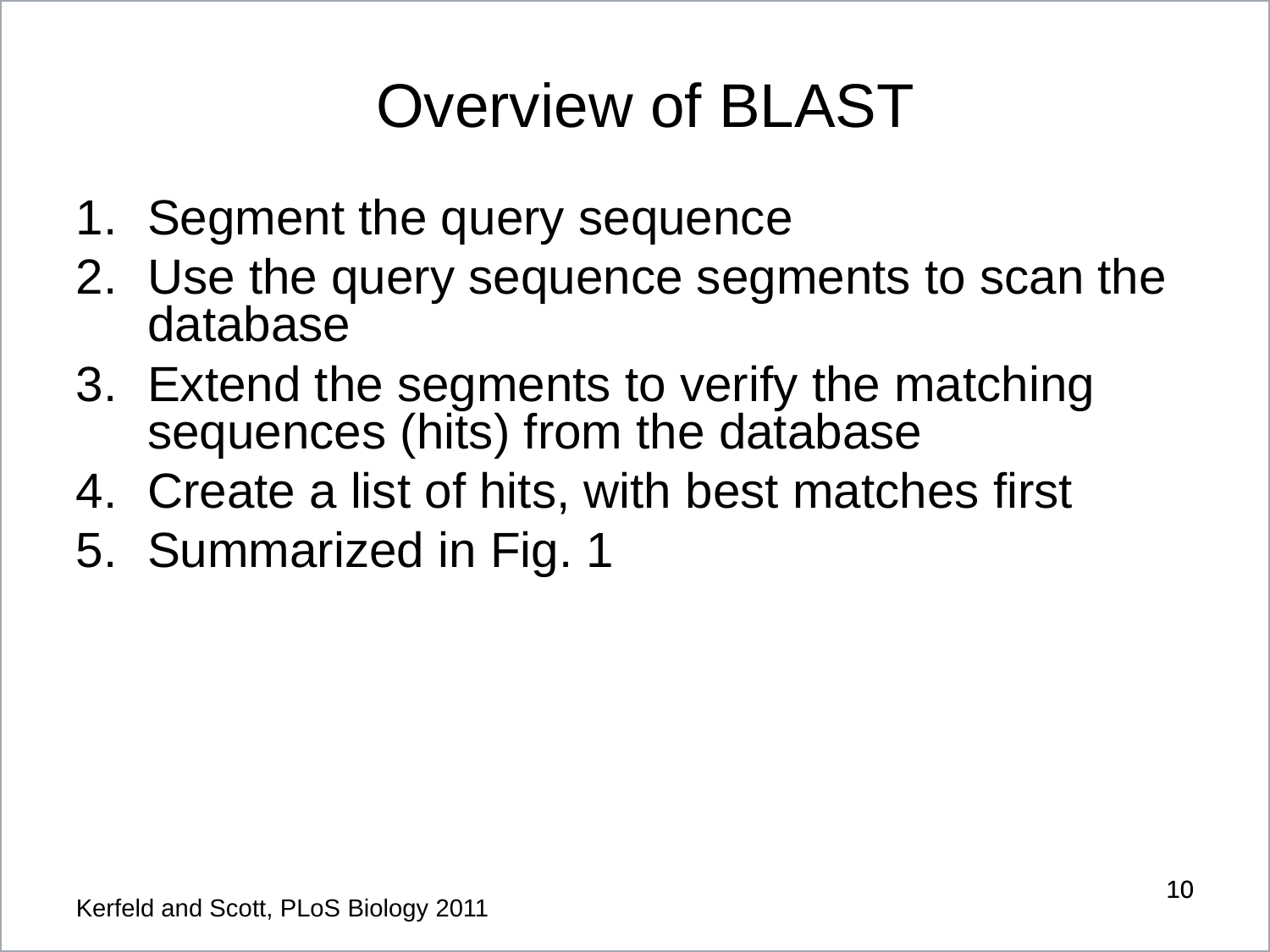

# Overview of BLAST
Segment the query sequence
Use the query sequence segments to scan the database
Extend the segments to verify the matching sequences (hits) from the database
Create a list of hits, with best matches first
Summarized in Fig. 1
<number>
<number>
Kerfeld and Scott, PLoS Biology 2011

## Slide 11
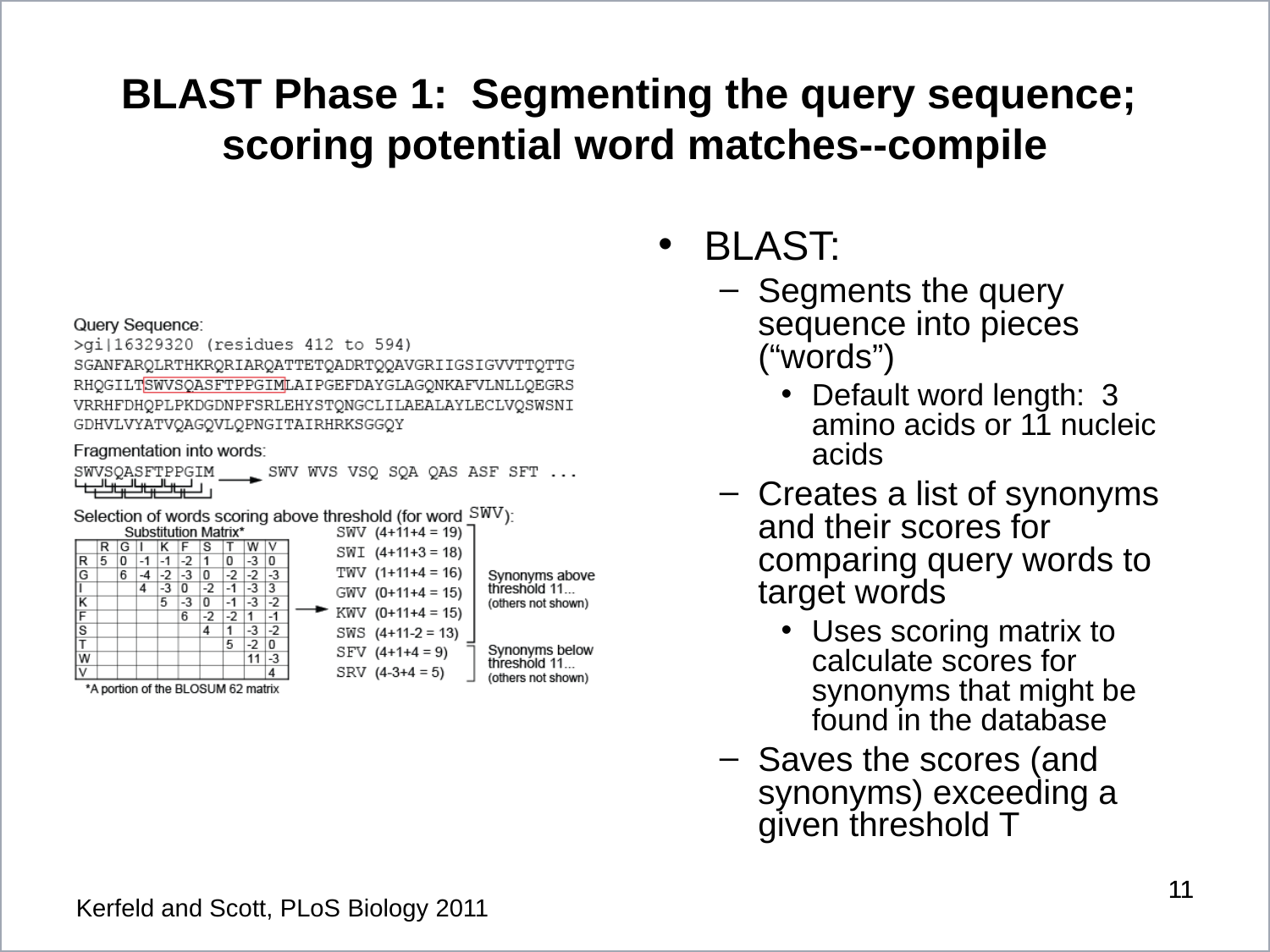

# BLAST Phase 1: Segmenting the query sequence; scoring potential word matches--compile
BLAST:
Segments the query sequence into pieces (“words”)
Default word length: 3 amino acids or 11 nucleic acids
Creates a list of synonyms and their scores for comparing query words to target words
Uses scoring matrix to calculate scores for synonyms that might be found in the database
Saves the scores (and synonyms) exceeding a given threshold T
<number>
<number>
Kerfeld and Scott, PLoS Biology 2011

## Slide 12
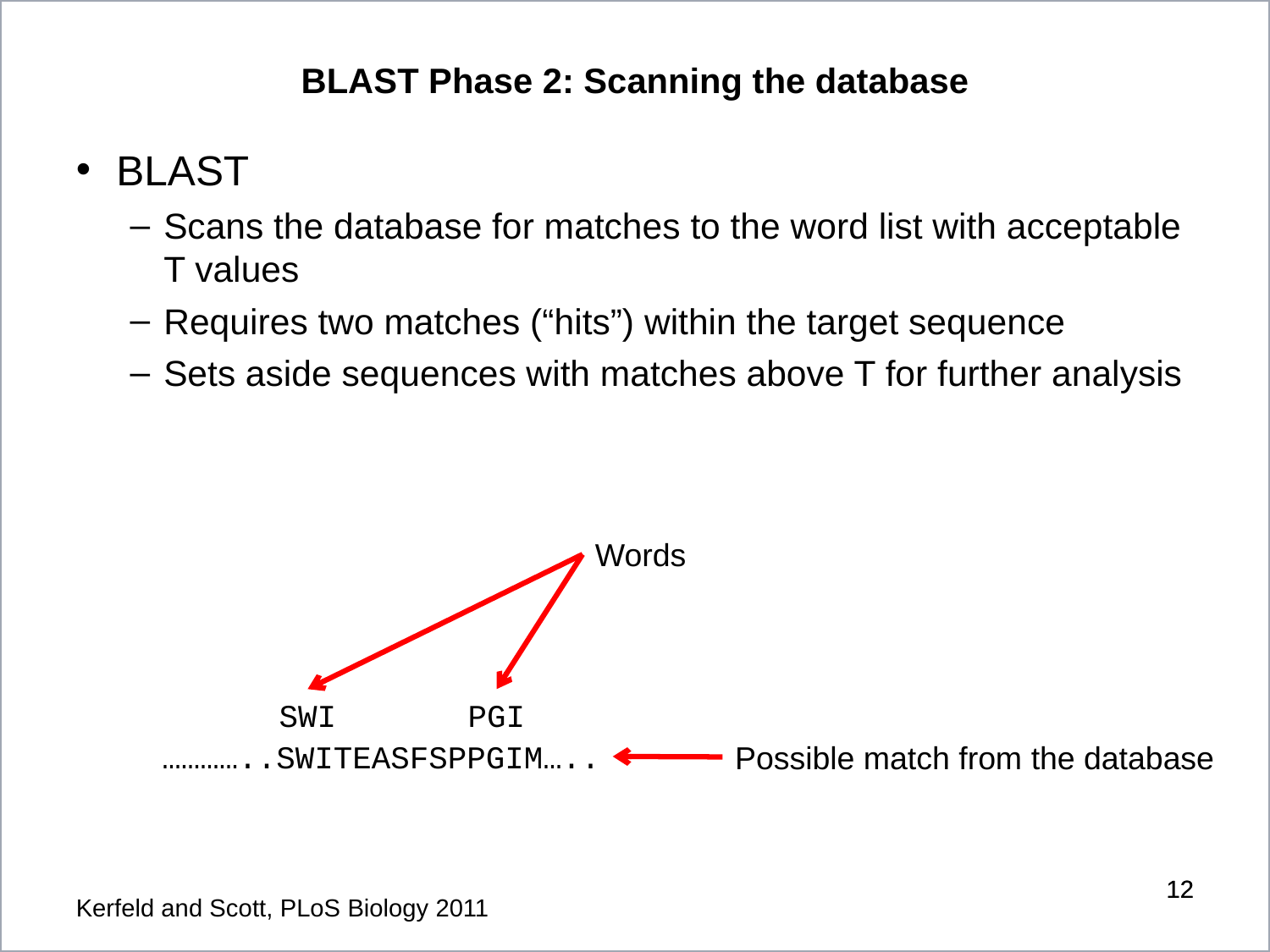

# BLAST Phase 2: Scanning the database
BLAST
Scans the database for matches to the word list with acceptable T values
Requires two matches (“hits”) within the target sequence
Sets aside sequences with matches above T for further analysis
Words
SWI
PGI
…………..SWITEASFSPPGIM…..
Possible match from the database
<number>
<number>
Kerfeld and Scott, PLoS Biology 2011

## Slide 13
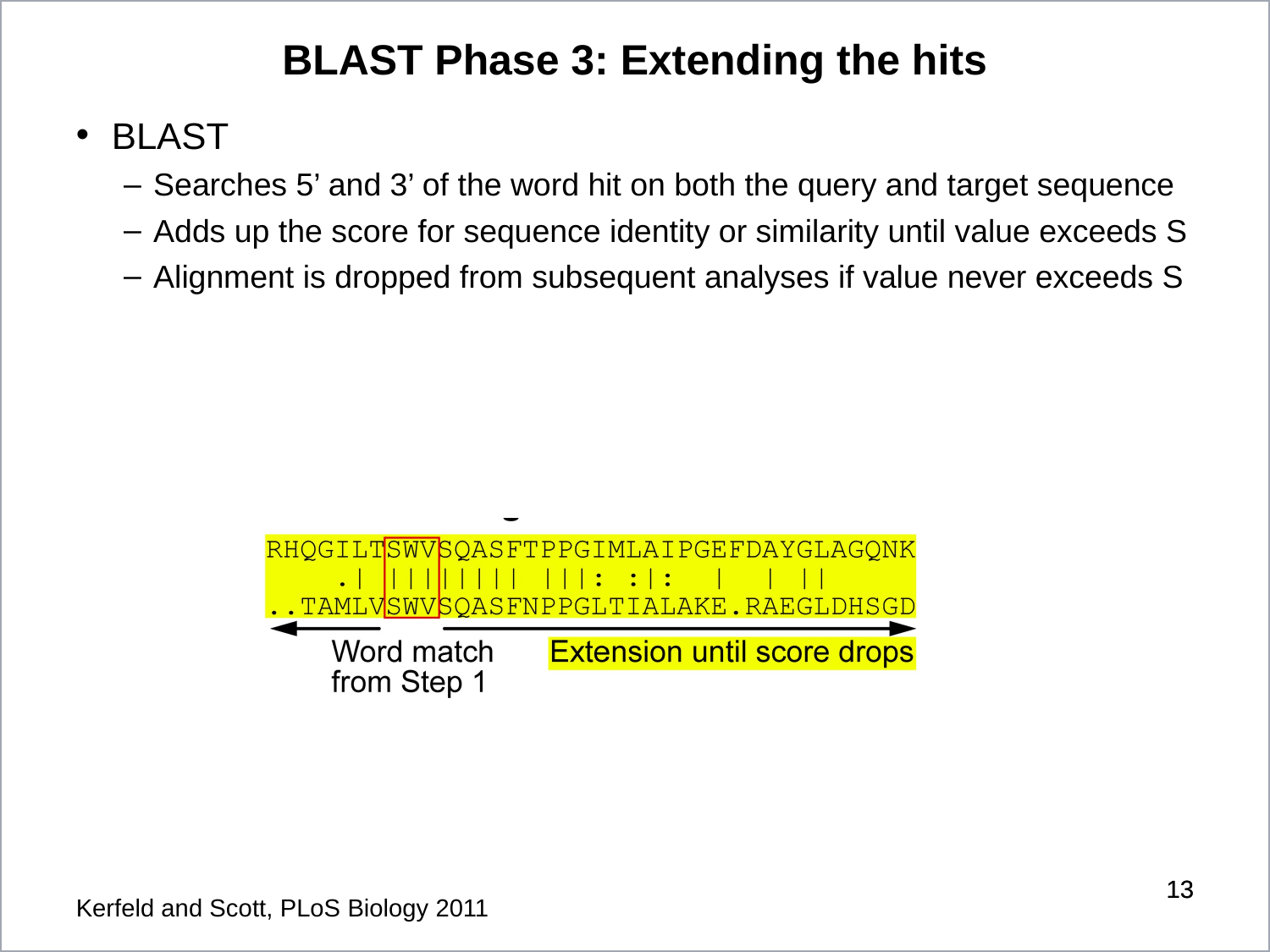

# BLAST Phase 3: Extending the hits
BLAST
Searches 5’ and 3’ of the word hit on both the query and target sequence
Adds up the score for sequence identity or similarity until value exceeds S
Alignment is dropped from subsequent analyses if value never exceeds S
<number>
<number>
Kerfeld and Scott, PLoS Biology 2011

## Slide 14
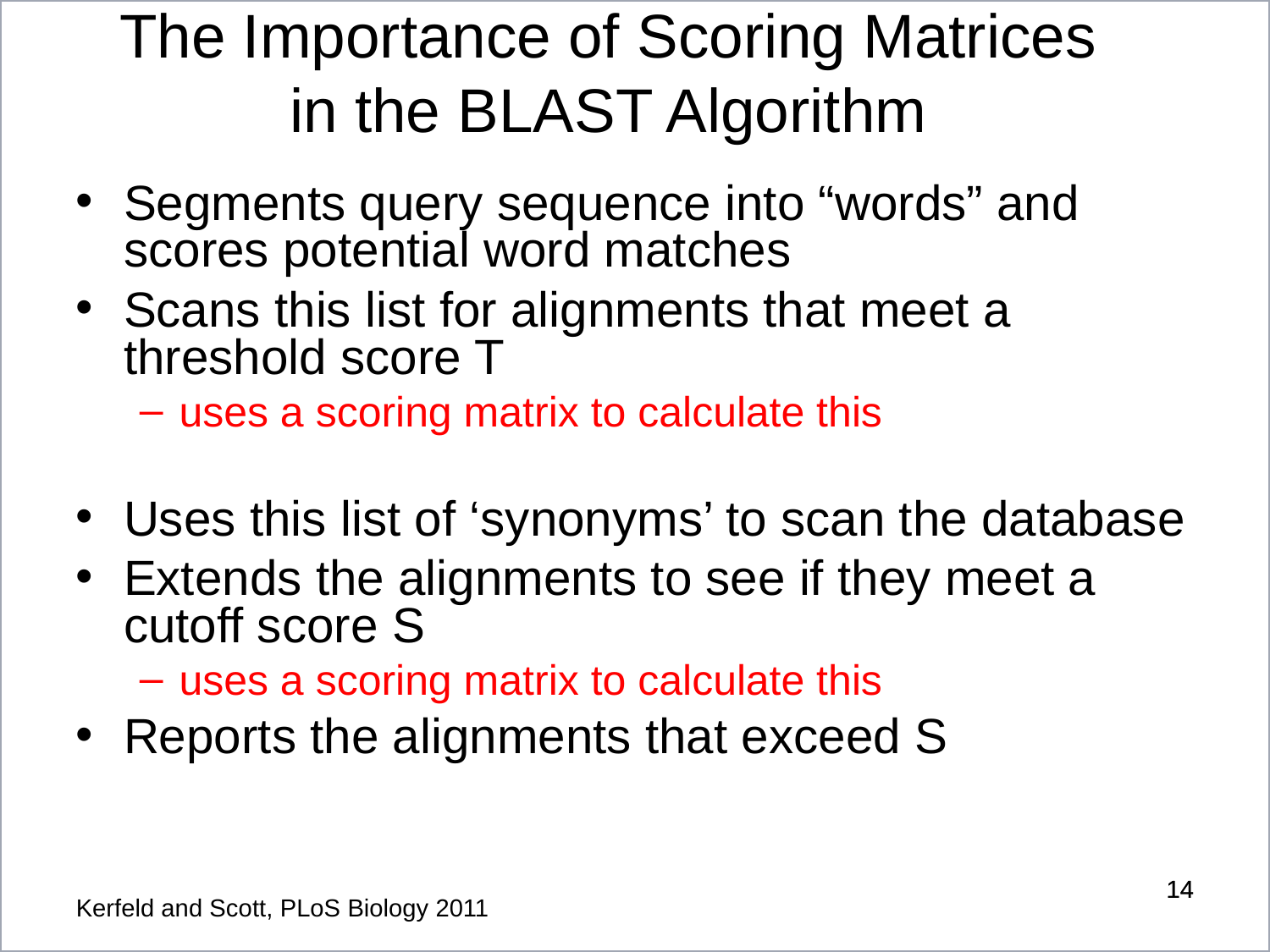

# The Importance of Scoring Matrices in the BLAST Algorithm
Segments query sequence into “words” and scores potential word matches
Scans this list for alignments that meet a threshold score T
uses a scoring matrix to calculate this
Uses this list of ‘synonyms’ to scan the database
Extends the alignments to see if they meet a cutoff score S
uses a scoring matrix to calculate this
Reports the alignments that exceed S
<number>
<number>
Kerfeld and Scott, PLoS Biology 2011

## Slide 15
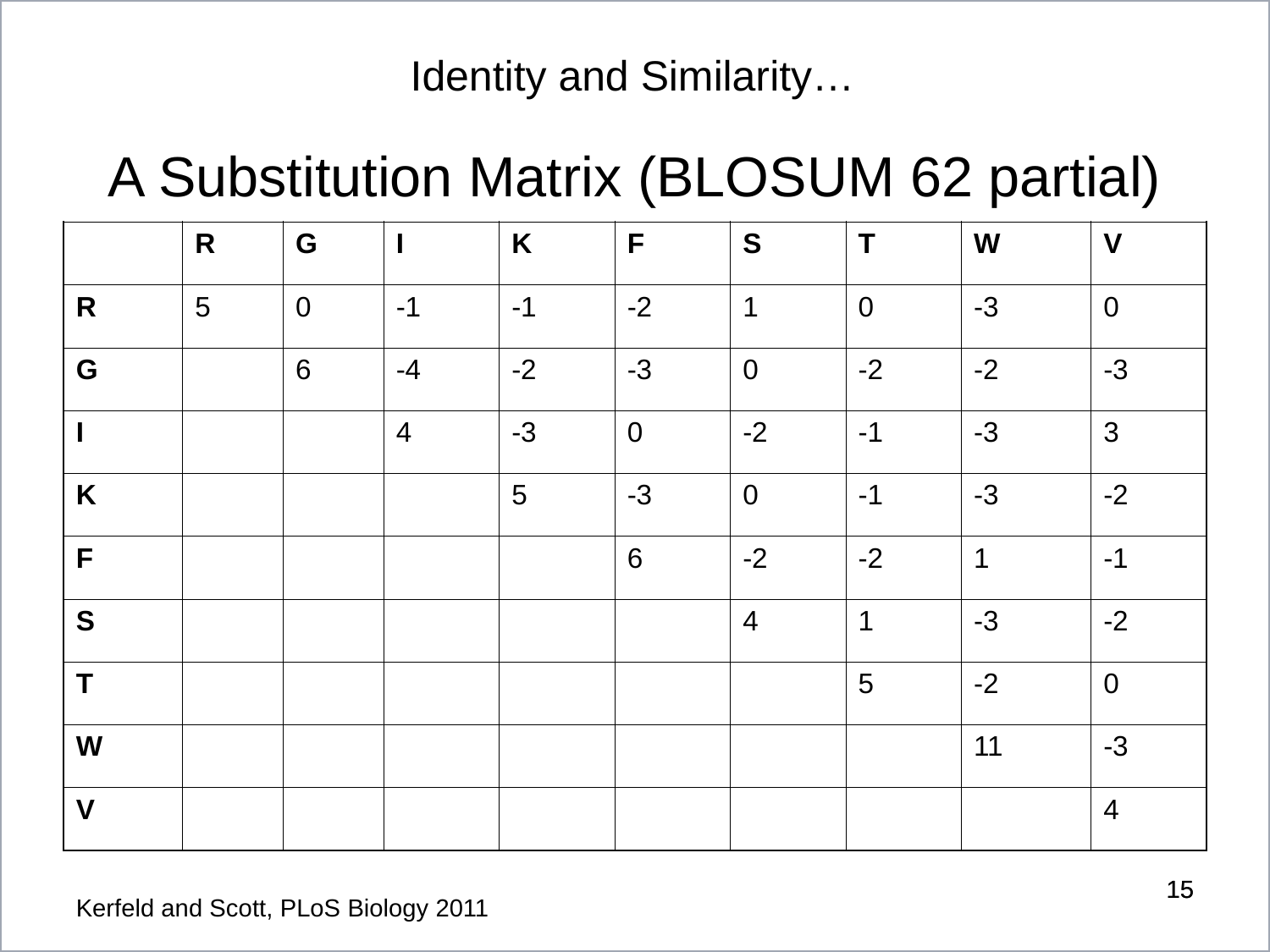

Identity and Similarity…
# A Substitution Matrix (BLOSUM 62 partial)
| | R | G | I | K | F | S | T | W | V |
| --- | --- | --- | --- | --- | --- | --- | --- | --- | --- |
| R | 5 | 0 | -1 | -1 | -2 | 1 | 0 | -3 | 0 |
| G | | 6 | -4 | -2 | -3 | 0 | -2 | -2 | -3 |
| I | | | 4 | -3 | 0 | -2 | -1 | -3 | 3 |
| K | | | | 5 | -3 | 0 | -1 | -3 | -2 |
| F | | | | | 6 | -2 | -2 | 1 | -1 |
| S | | | | | | 4 | 1 | -3 | -2 |
| T | | | | | | | 5 | -2 | 0 |
| W | | | | | | | | 11 | -3 |
| V | | | | | | | | | 4 |
<number>
<number>
Kerfeld and Scott, PLoS Biology 2011

## Slide 16
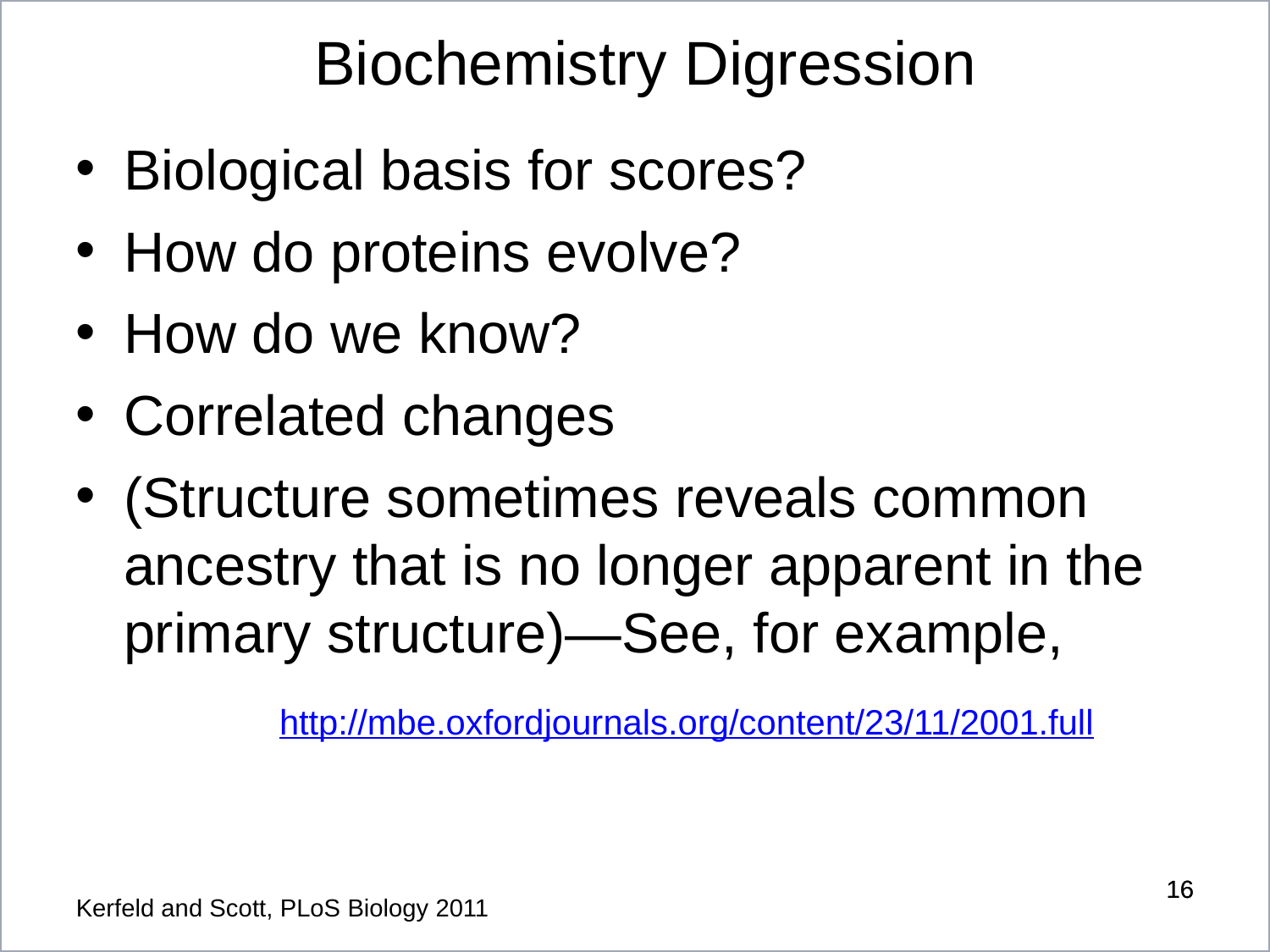

# Biochemistry Digression
Biological basis for scores?
How do proteins evolve?
How do we know?
Correlated changes
(Structure sometimes reveals common ancestry that is no longer apparent in the primary structure)—See, for example,
 http://mbe.oxfordjournals.org/content/23/11/2001.full
<number>
<number>
Kerfeld and Scott, PLoS Biology 2011

## Slide 17
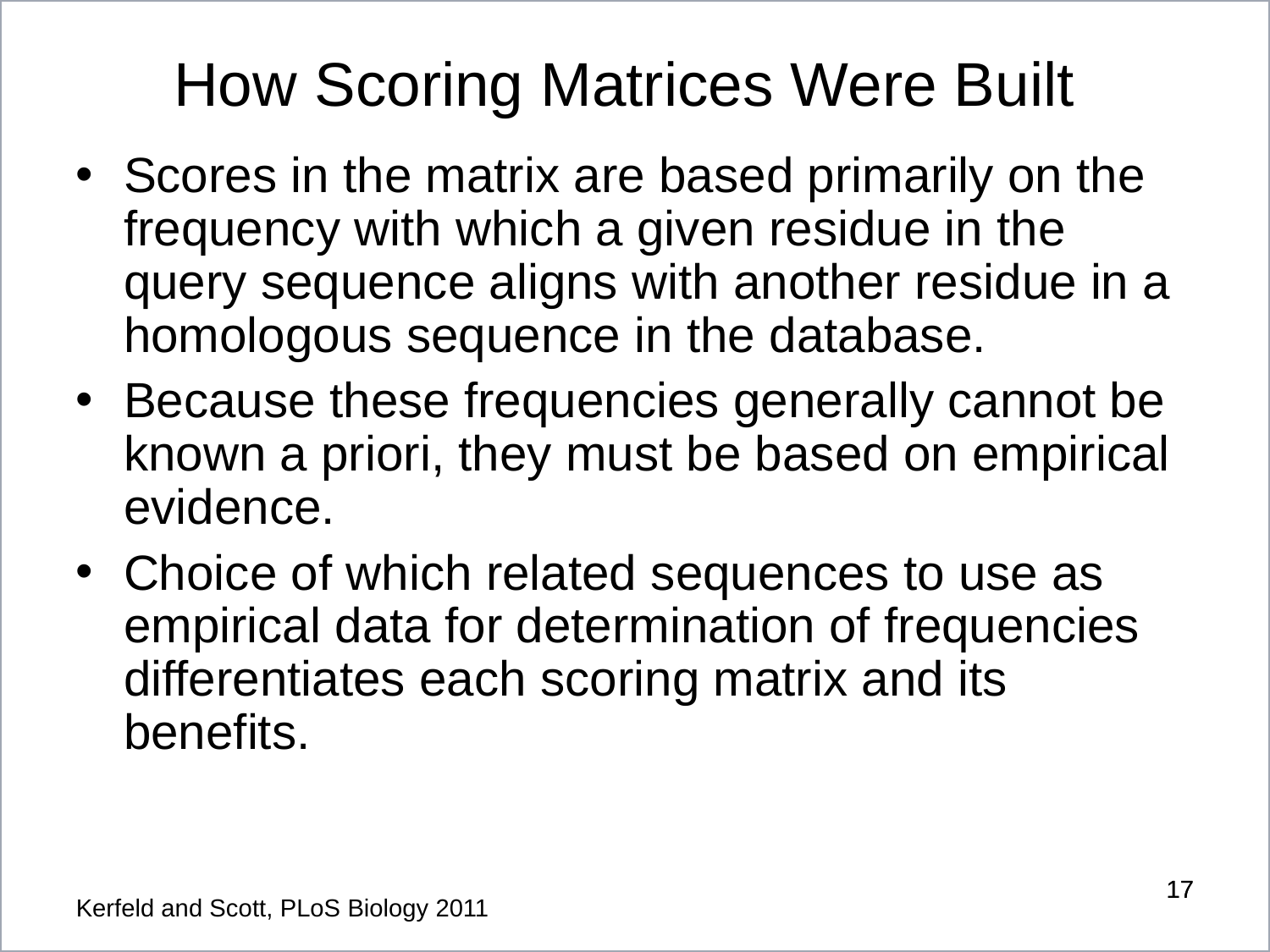

# How Scoring Matrices Were Built
Scores in the matrix are based primarily on the frequency with which a given residue in the query sequence aligns with another residue in a homologous sequence in the database.
Because these frequencies generally cannot be known a priori, they must be based on empirical evidence.
Choice of which related sequences to use as empirical data for determination of frequencies differentiates each scoring matrix and its benefits.
<number>
<number>
Kerfeld and Scott, PLoS Biology 2011

## Slide 18
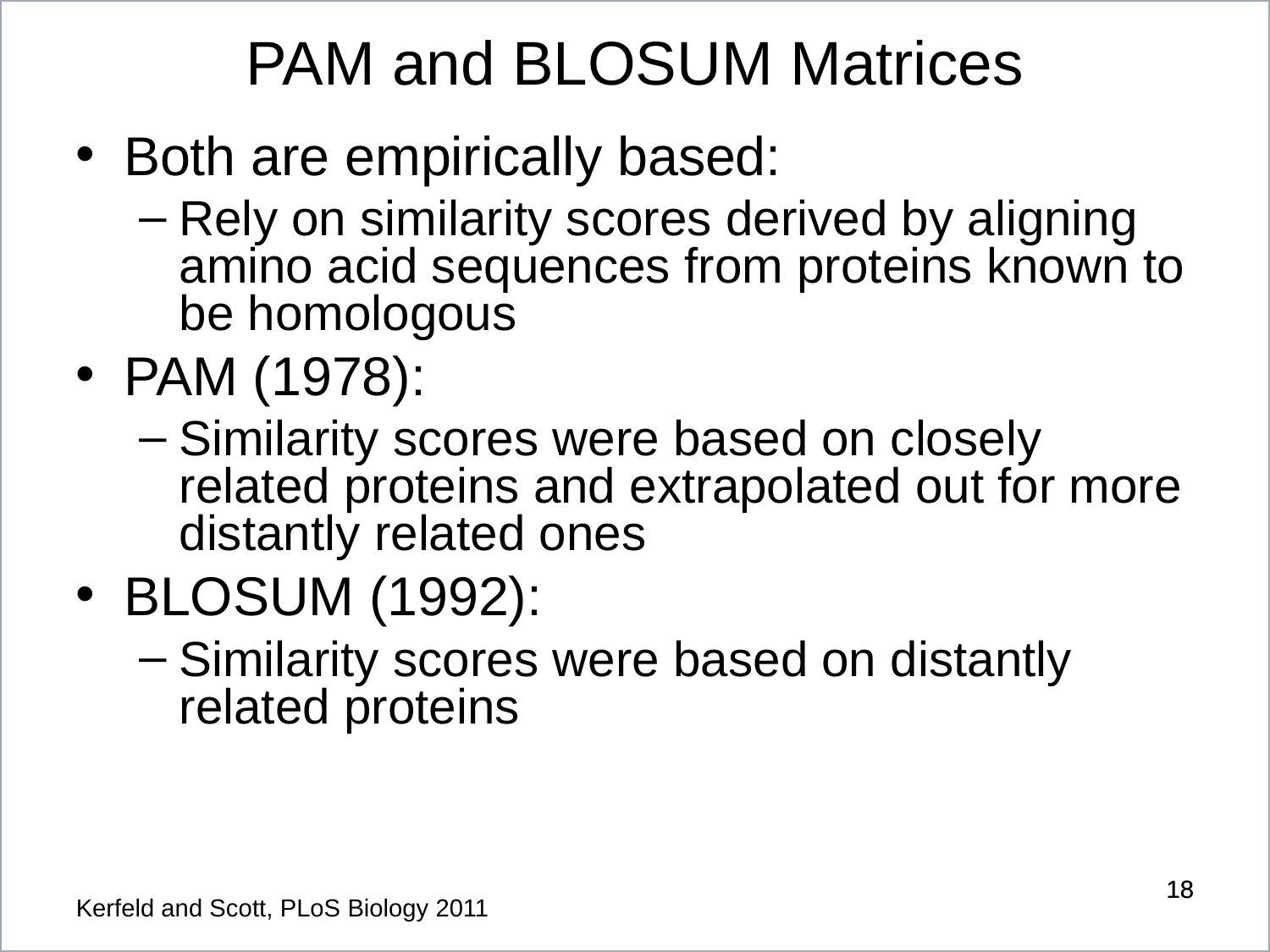

# PAM and BLOSUM Matrices
Both are empirically based:
Rely on similarity scores derived by aligning amino acid sequences from proteins known to be homologous
PAM (1978):
Similarity scores were based on closely related proteins and extrapolated out for more distantly related ones
BLOSUM (1992):
Similarity scores were based on distantly related proteins
<number>
<number>
Kerfeld and Scott, PLoS Biology 2011

## Slide 19
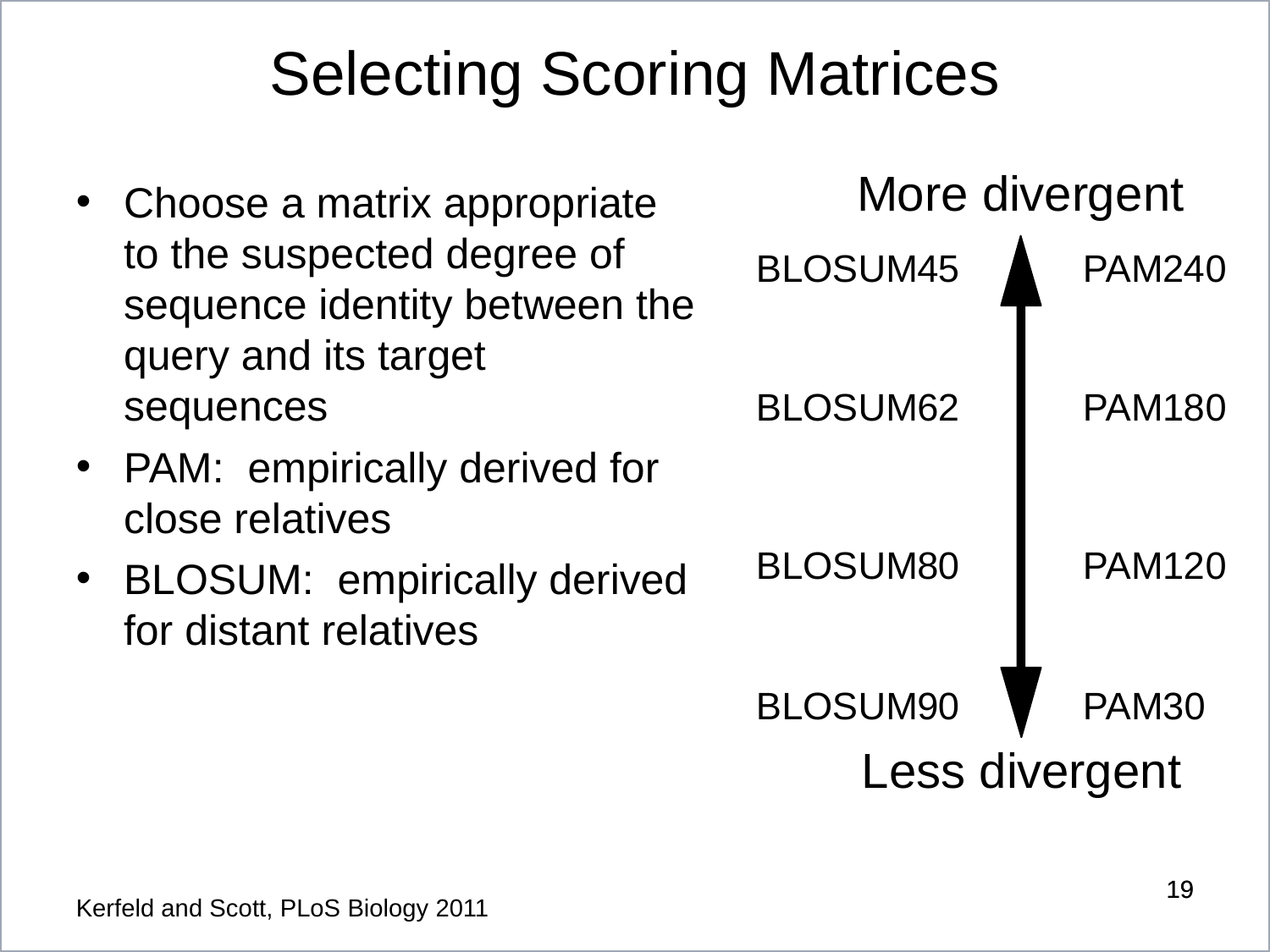

# Selecting Scoring Matrices
Choose a matrix appropriate to the suspected degree of sequence identity between the query and its target sequences
PAM: empirically derived for close relatives
BLOSUM: empirically derived for distant relatives
<number>
<number>
Kerfeld and Scott, PLoS Biology 2011

## Slide 20
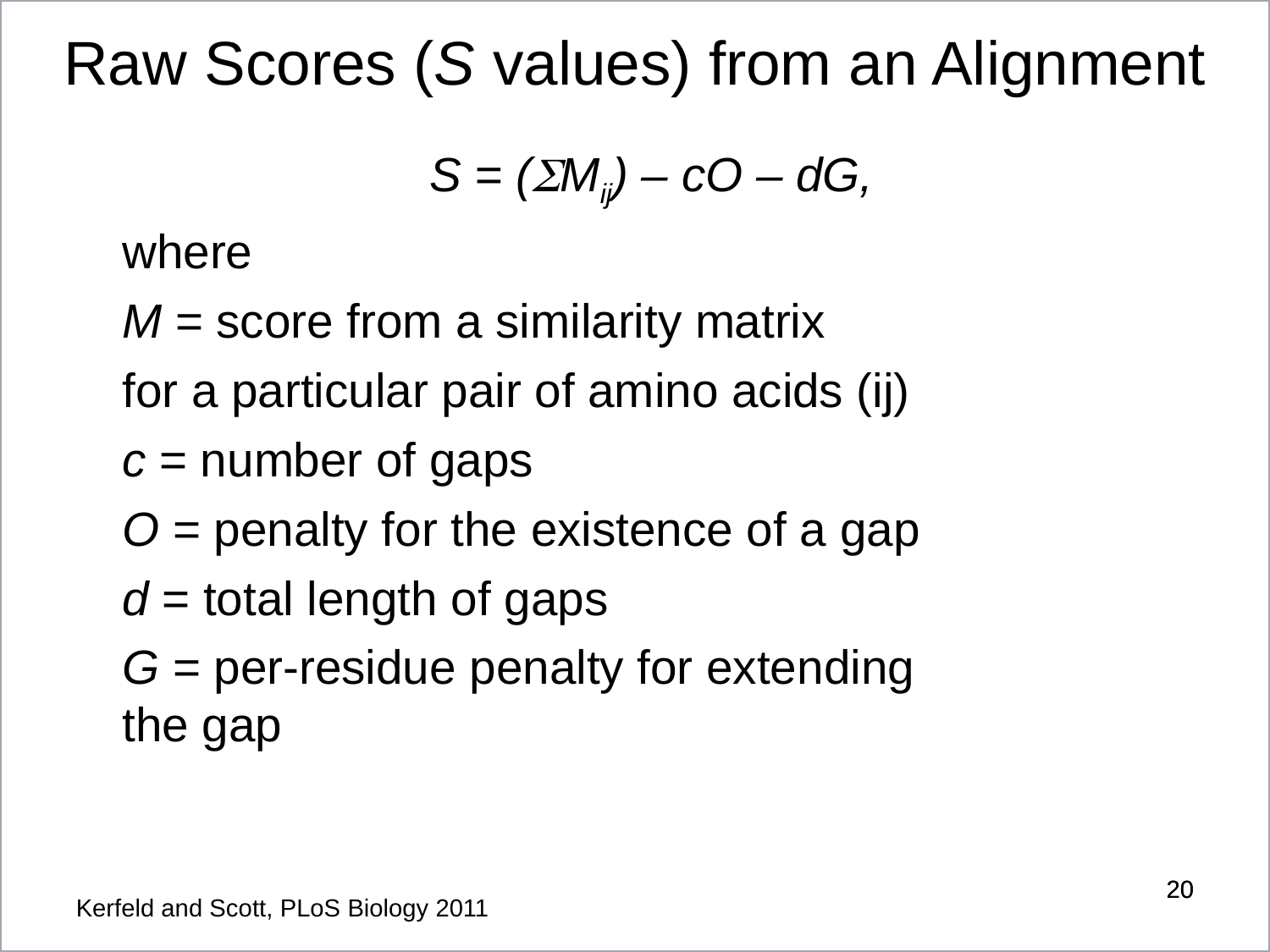

# Raw Scores (S values) from an Alignment
S = (Mij) – cO – dG,
	where
		M = score from a similarity matrix
			for a particular pair of amino acids (ij)
		c = number of gaps
		O = penalty for the existence of a gap
		d = total length of gaps
		G = per-residue penalty for extending
		the gap
<number>
<number>
Kerfeld and Scott, PLoS Biology 2011

## Slide 21
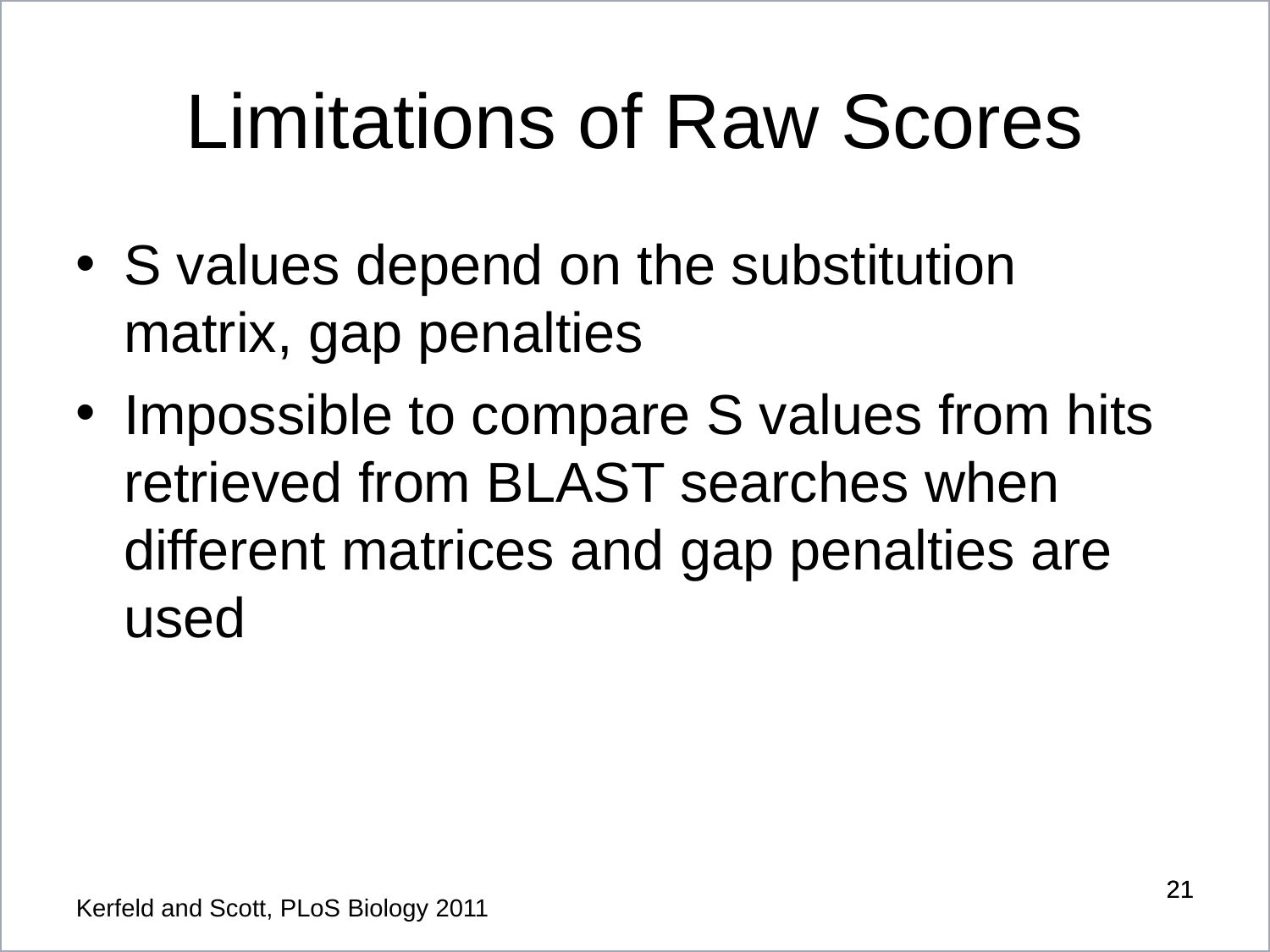

# Limitations of Raw Scores
S values depend on the substitution matrix, gap penalties
Impossible to compare S values from hits retrieved from BLAST searches when different matrices and gap penalties are used
<number>
<number>
Kerfeld and Scott, PLoS Biology 2011

## Slide 22
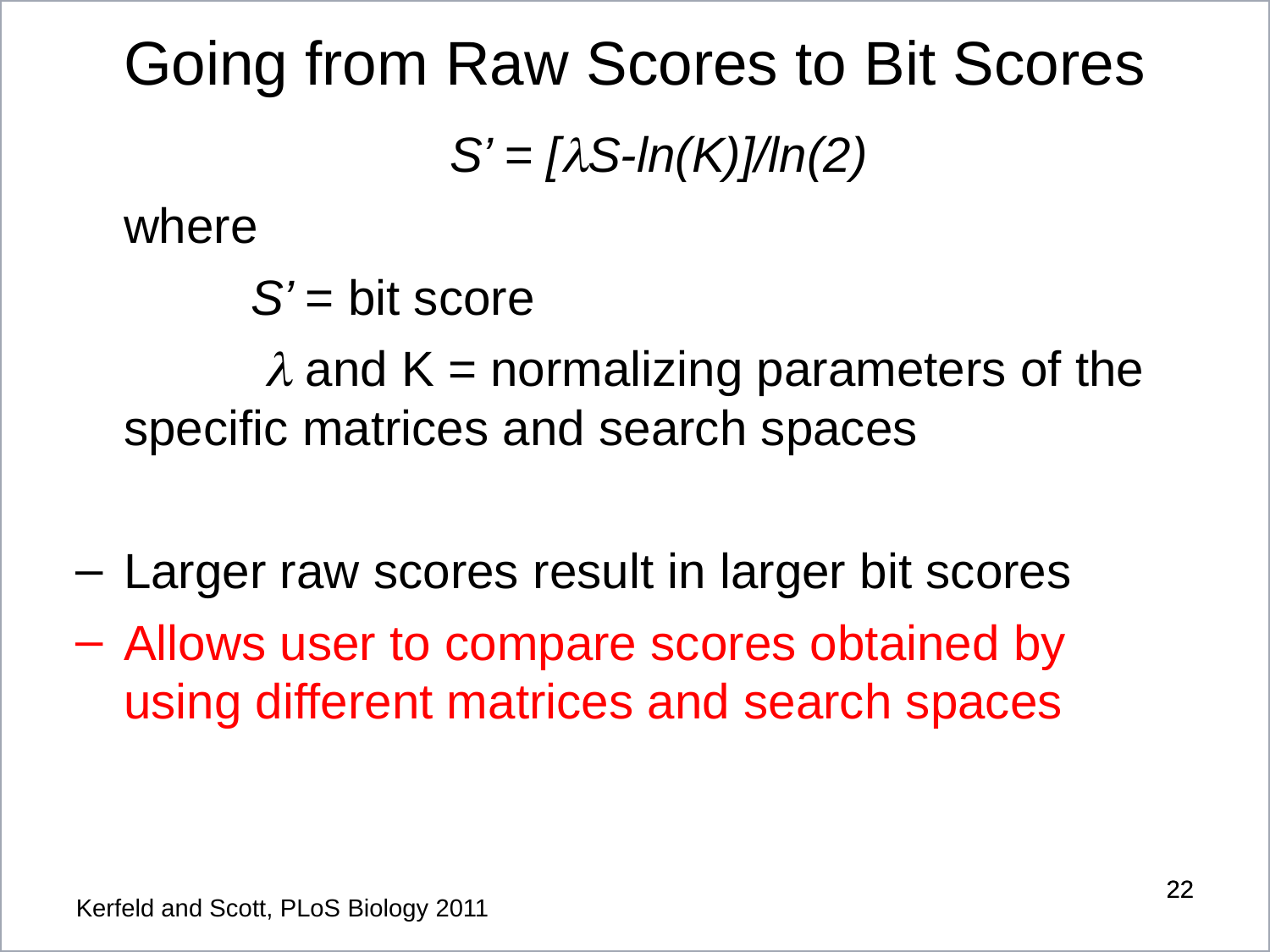

# Going from Raw Scores to Bit Scores
S’ = [S-ln(K)]/ln(2)
where
	S’ = bit score
	  and K = normalizing parameters of the specific matrices and search spaces
Larger raw scores result in larger bit scores
Allows user to compare scores obtained by using different matrices and search spaces
<number>
<number>
Kerfeld and Scott, PLoS Biology 2011

## Slide 23
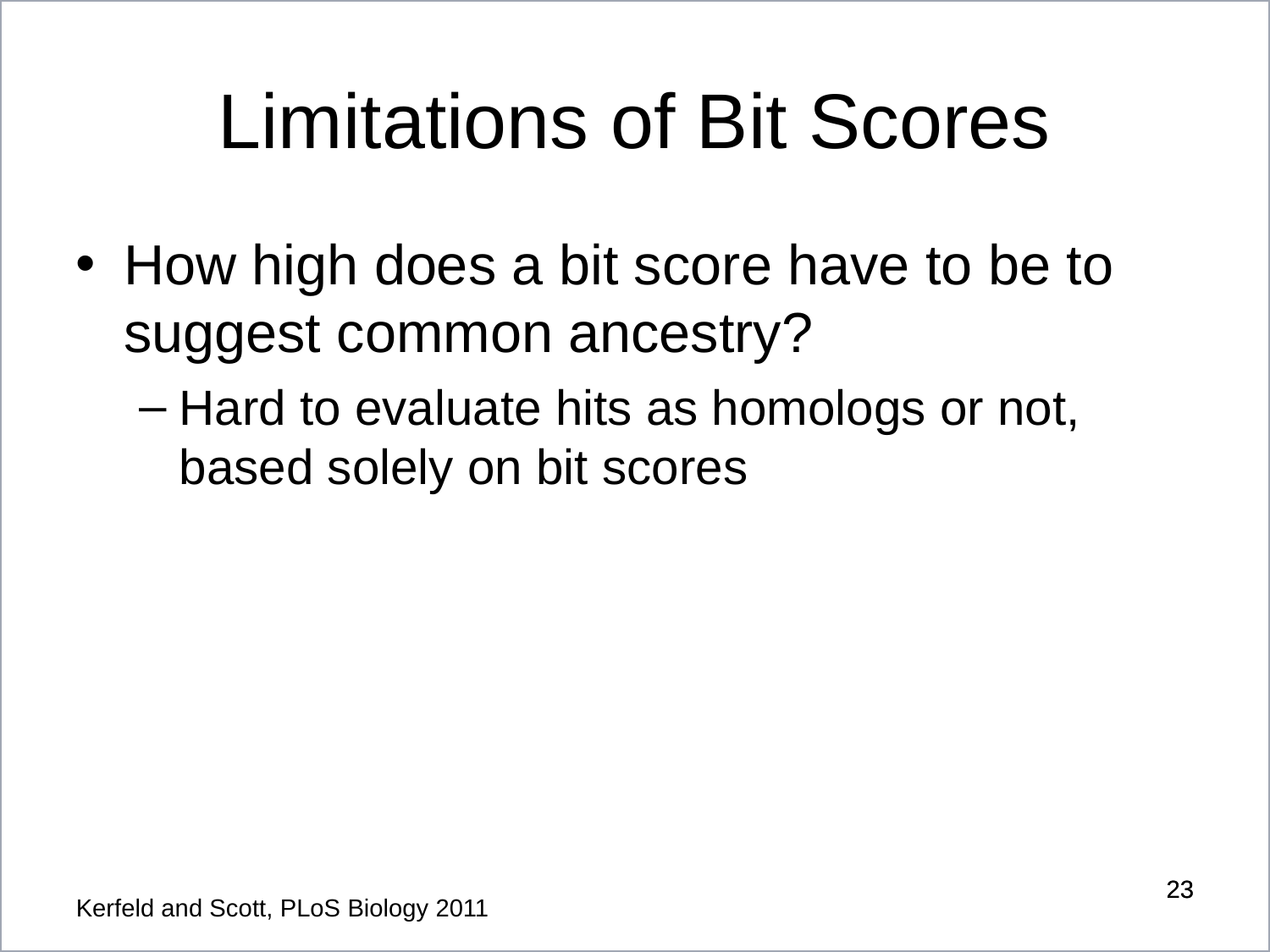

# Limitations of Bit Scores
How high does a bit score have to be to suggest common ancestry?
Hard to evaluate hits as homologs or not, based solely on bit scores
<number>
<number>
Kerfeld and Scott, PLoS Biology 2011

## Slide 24
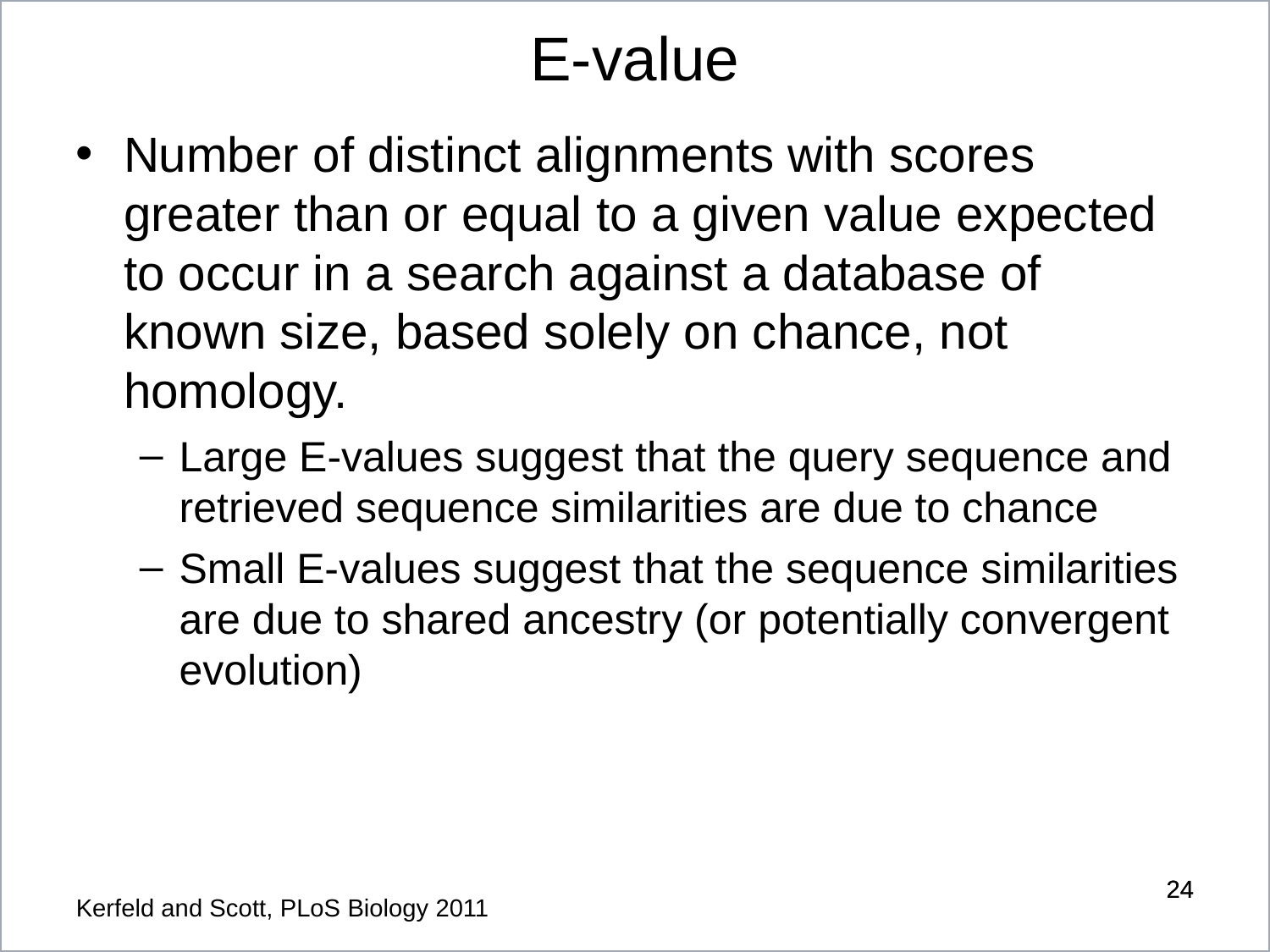

# E-value
Number of distinct alignments with scores greater than or equal to a given value expected to occur in a search against a database of known size, based solely on chance, not homology.
Large E-values suggest that the query sequence and retrieved sequence similarities are due to chance
Small E-values suggest that the sequence similarities are due to shared ancestry (or potentially convergent evolution)
<number>
<number>
Kerfeld and Scott, PLoS Biology 2011

## Slide 25
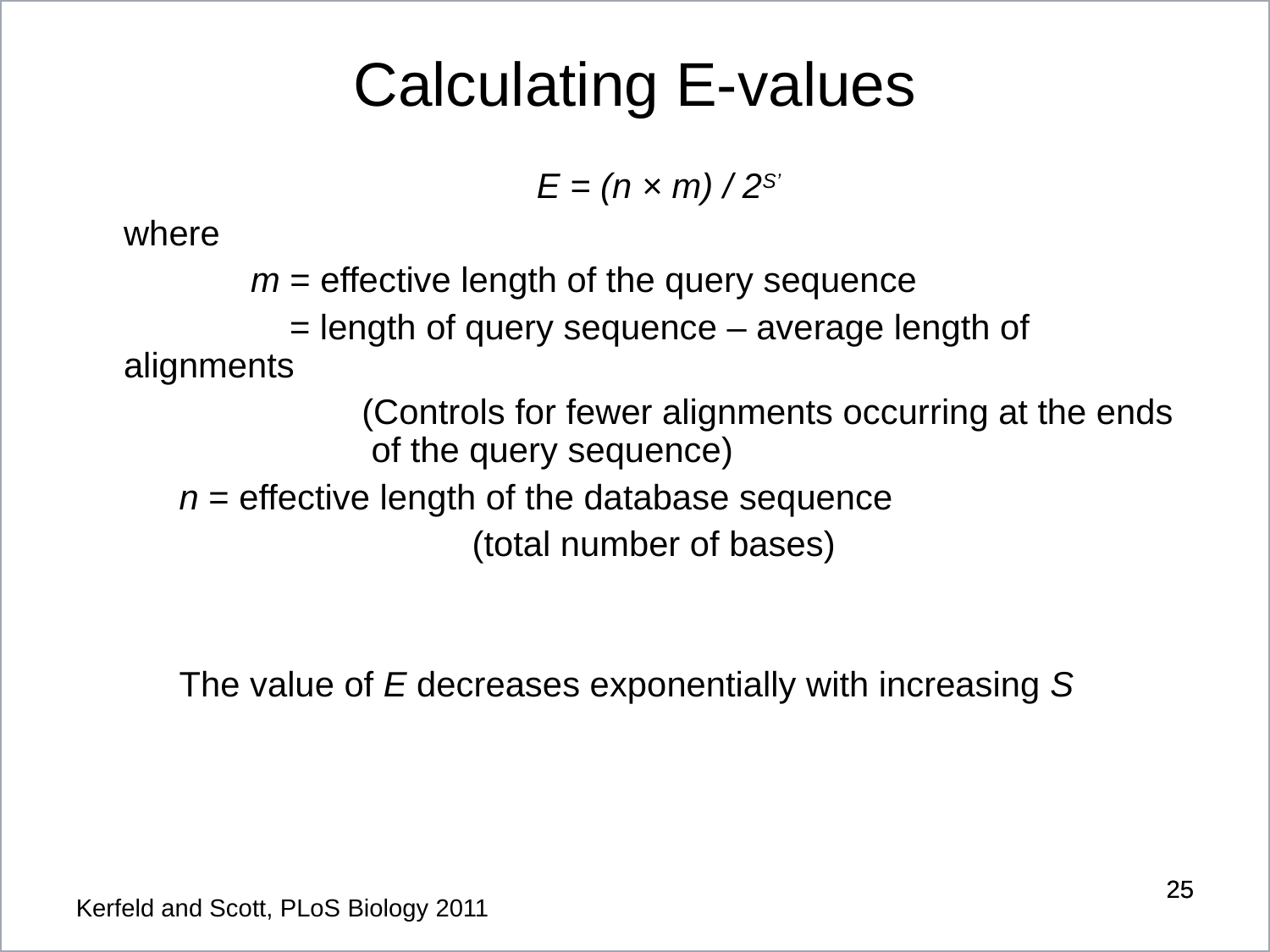

# Calculating E-values
E = (n × m) / 2S’
where
	m = effective length of the query sequence
	 = length of query sequence – average length of alignments
	(Controls for fewer alignments occurring at the ends
	 of the query sequence)
n = effective length of the database sequence
		 (total number of bases)
The value of E decreases exponentially with increasing S
<number>
<number>
Kerfeld and Scott, PLoS Biology 2011

## Slide 26
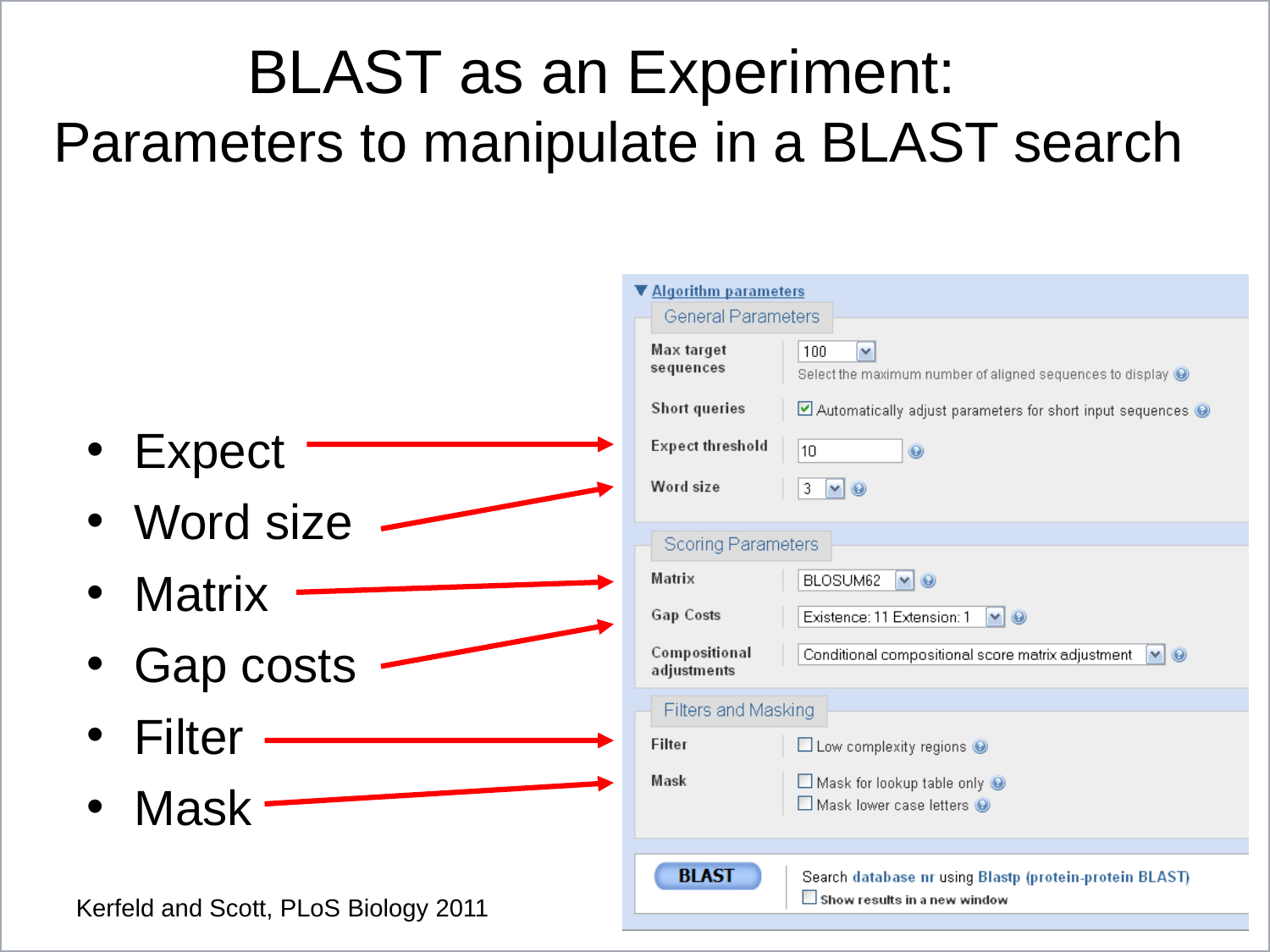

# BLAST as an Experiment: Parameters to manipulate in a BLAST search
Expect
Word size
Matrix
Gap costs
Filter
Mask
<number>
<number>
Kerfeld and Scott, PLoS Biology 2011

## Slide 27
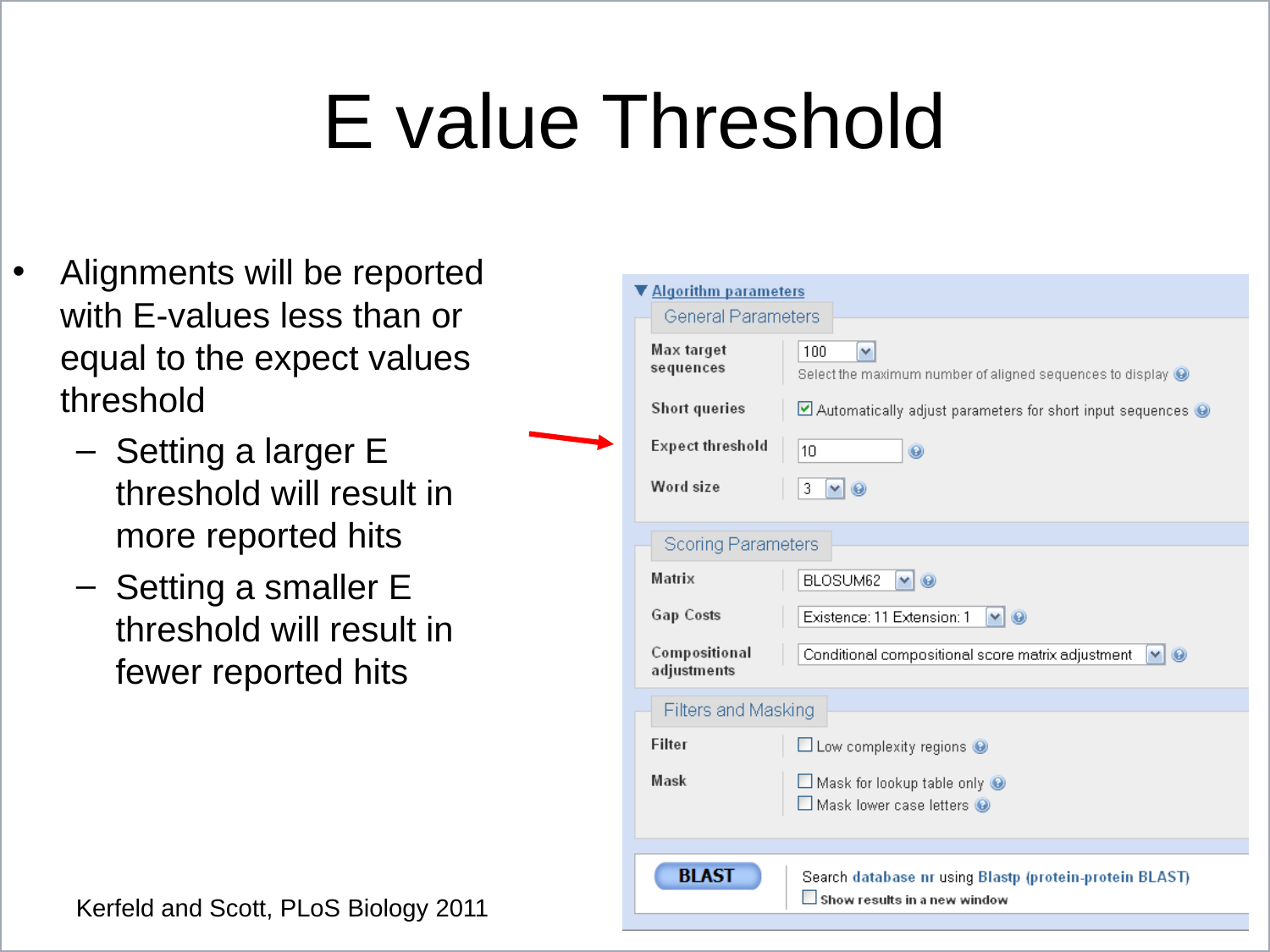

# E value Threshold
Alignments will be reported with E-values less than or equal to the expect values threshold
Setting a larger E threshold will result in more reported hits
Setting a smaller E threshold will result in fewer reported hits
<number>
<number>
Kerfeld and Scott, PLoS Biology 2011

## Slide 28
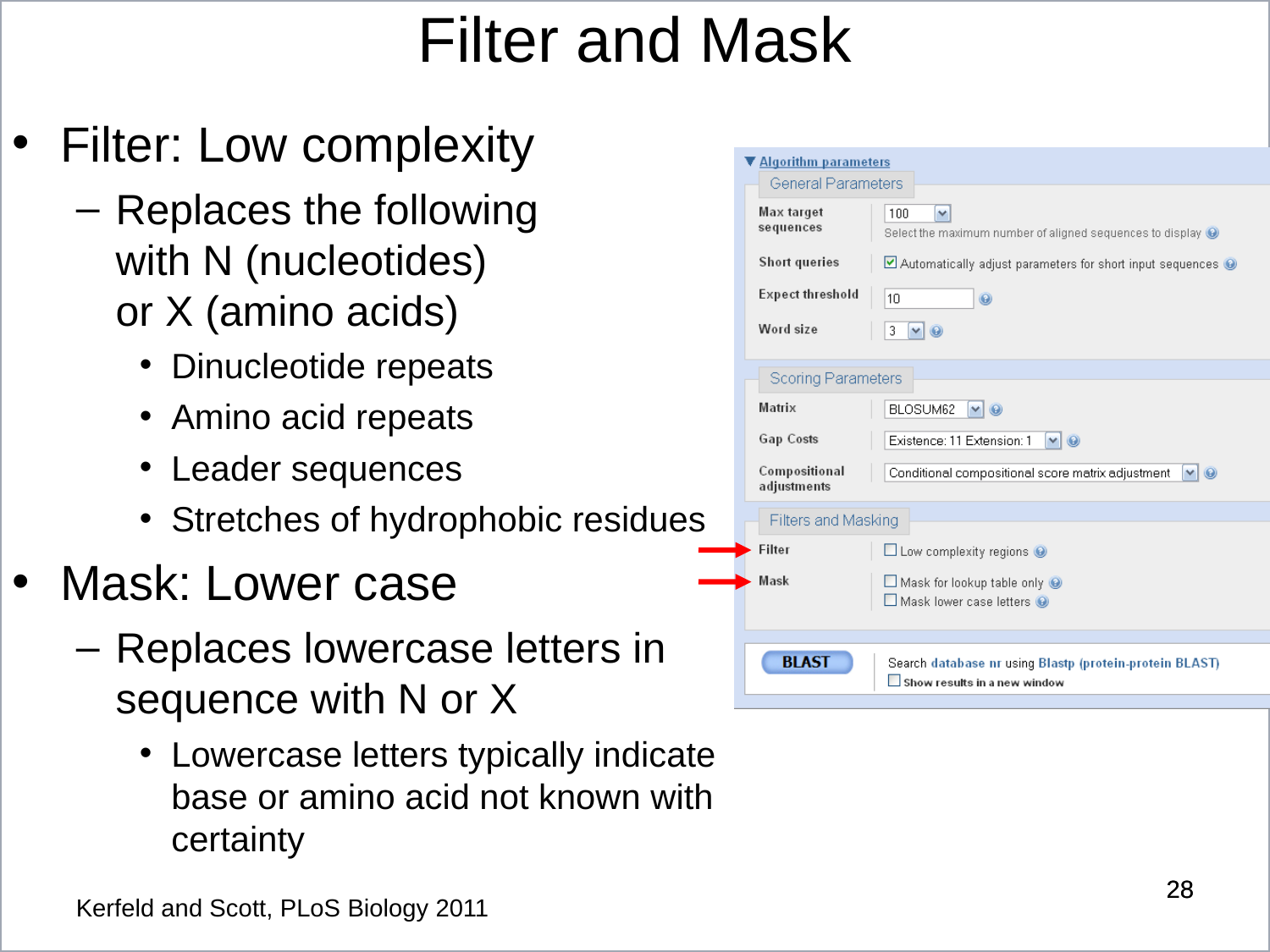

# Filter and Mask
Filter: Low complexity
Replaces the following
with N (nucleotides)
or X (amino acids)
Dinucleotide repeats
Amino acid repeats
Leader sequences
Stretches of hydrophobic residues
Mask: Lower case
Replaces lowercase letters in sequence with N or X
Lowercase letters typically indicate base or amino acid not known with certainty
<number>
<number>
Kerfeld and Scott, PLoS Biology 2011

## Slide 29
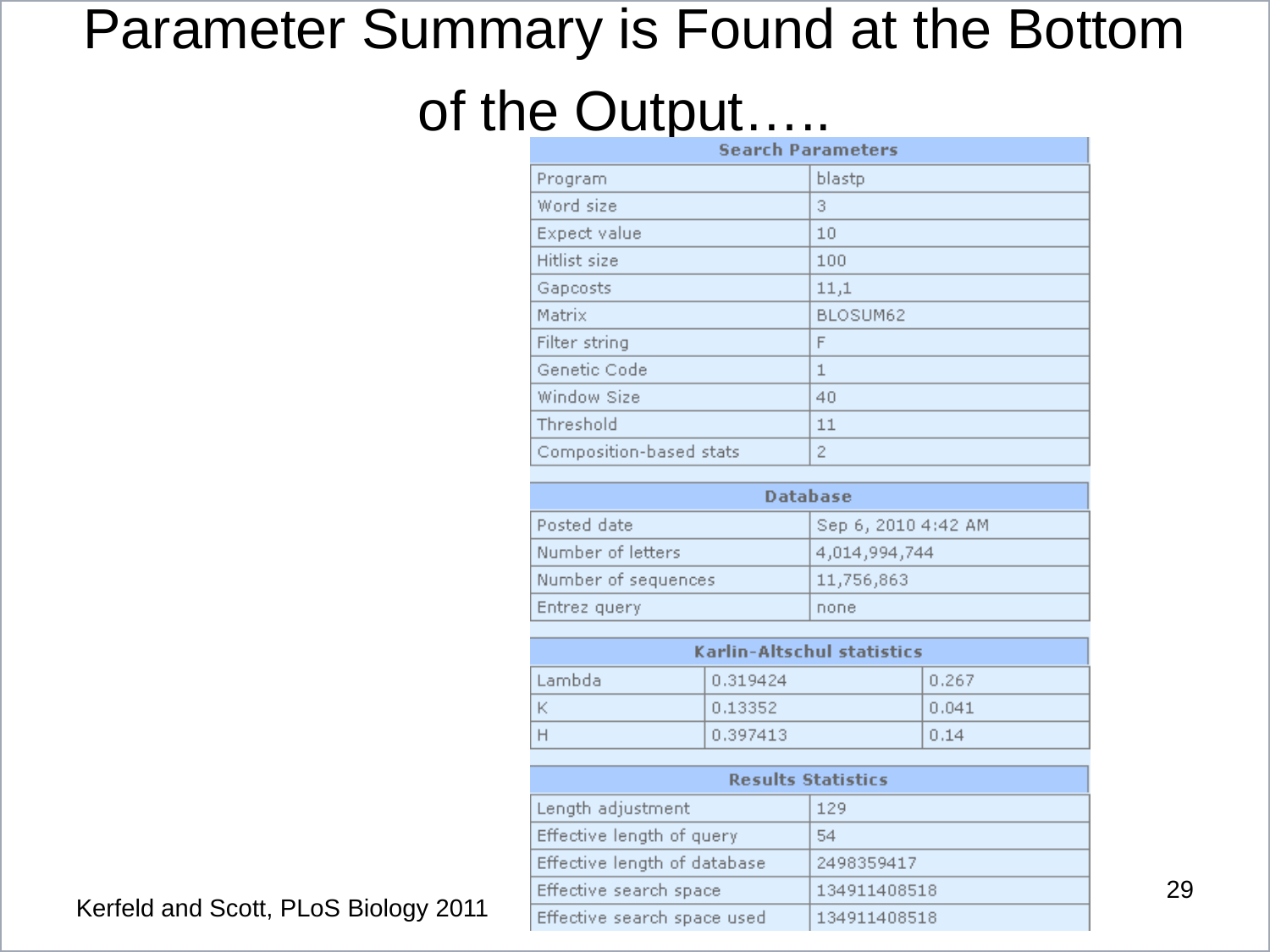

# Parameter Summary is Found at the Bottom of the Output…..
<number>
Kerfeld and Scott, PLoS Biology 2011

## Slide 30
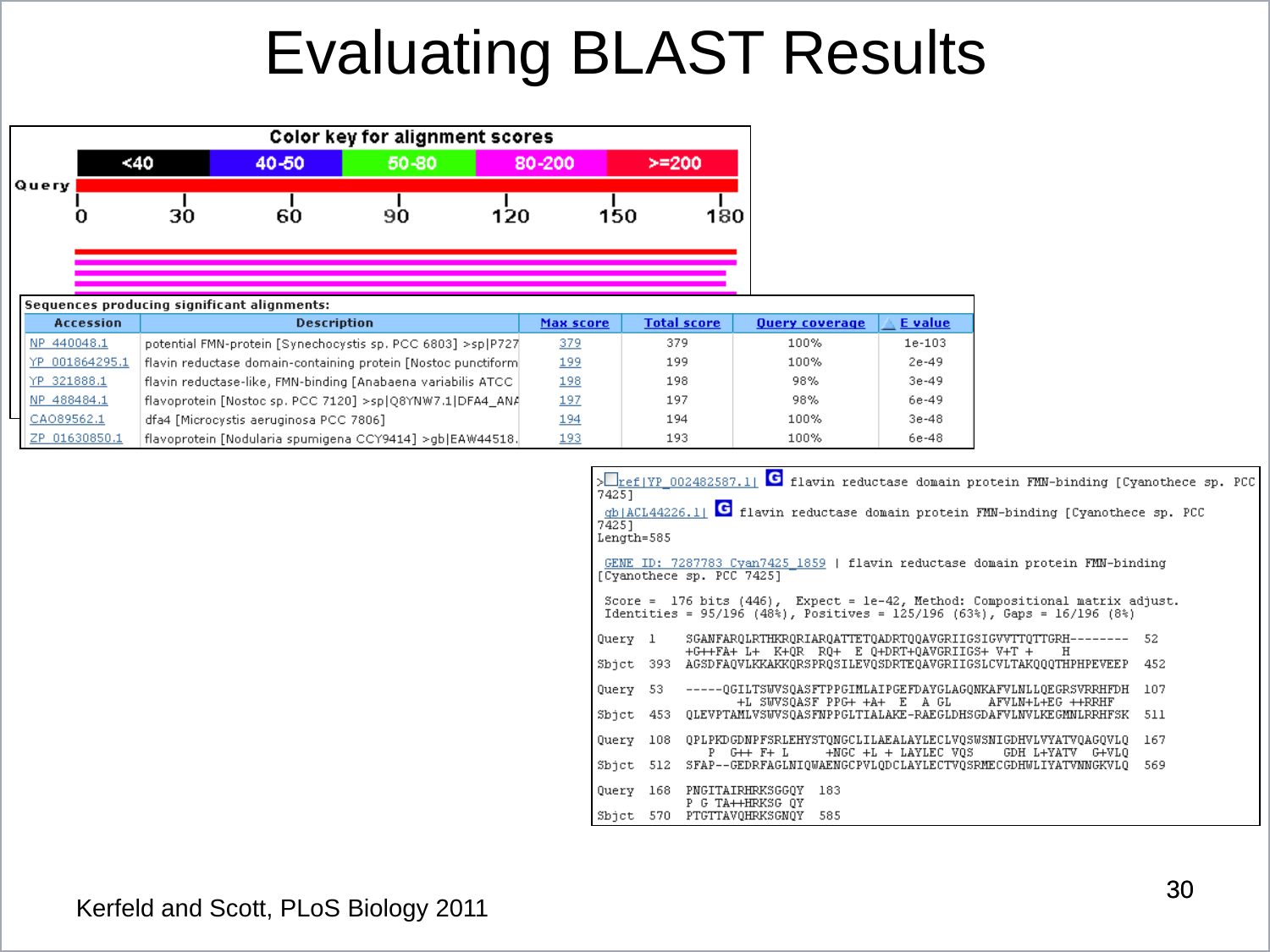

# Evaluating BLAST Results
<number>
<number>
Kerfeld and Scott, PLoS Biology 2011

## Slide 31
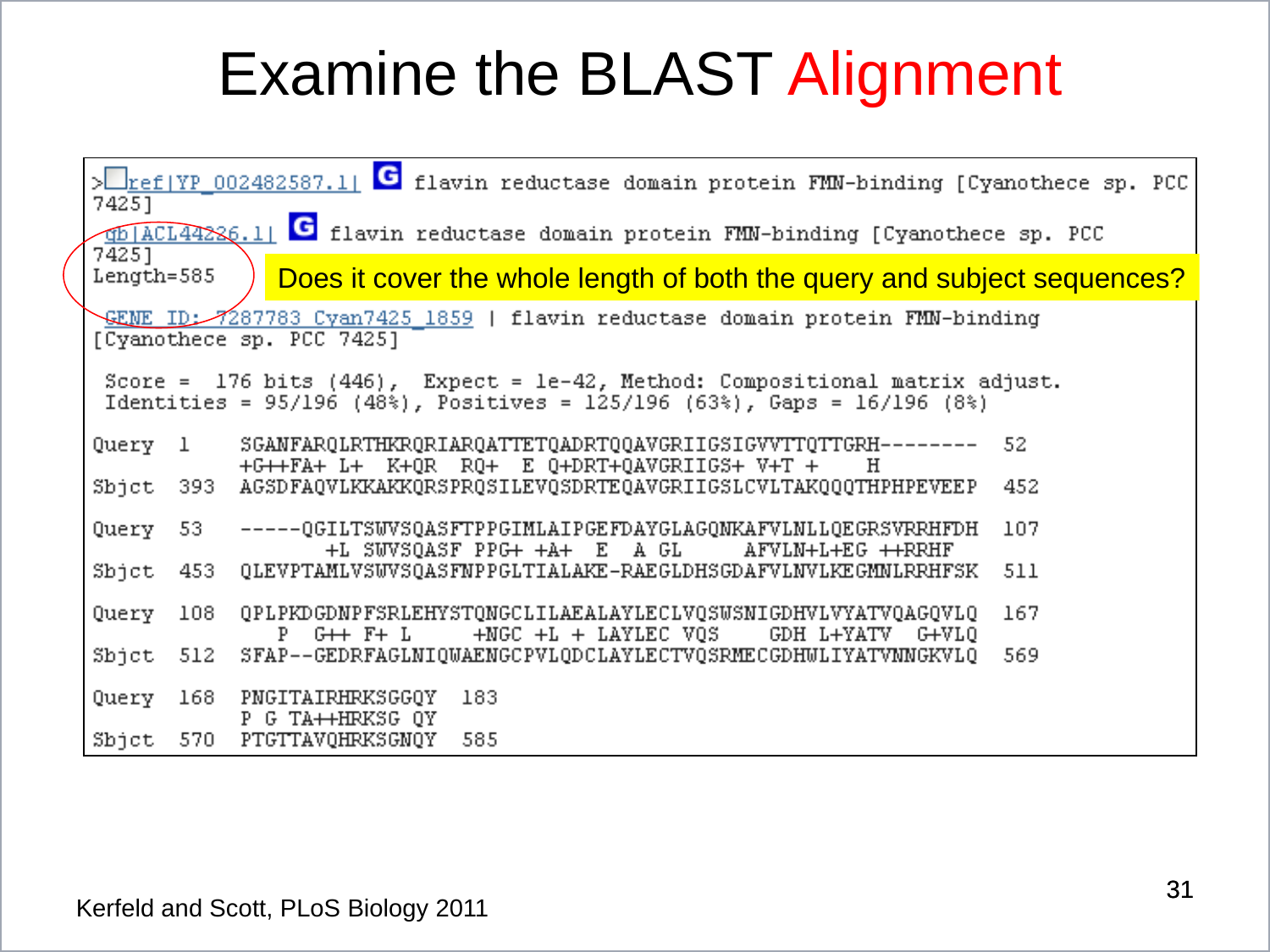

# Examine the BLAST Alignment
Does it cover the whole length of both the query and subject sequences?
<number>
<number>
Kerfeld and Scott, PLoS Biology 2011

## Slide 32
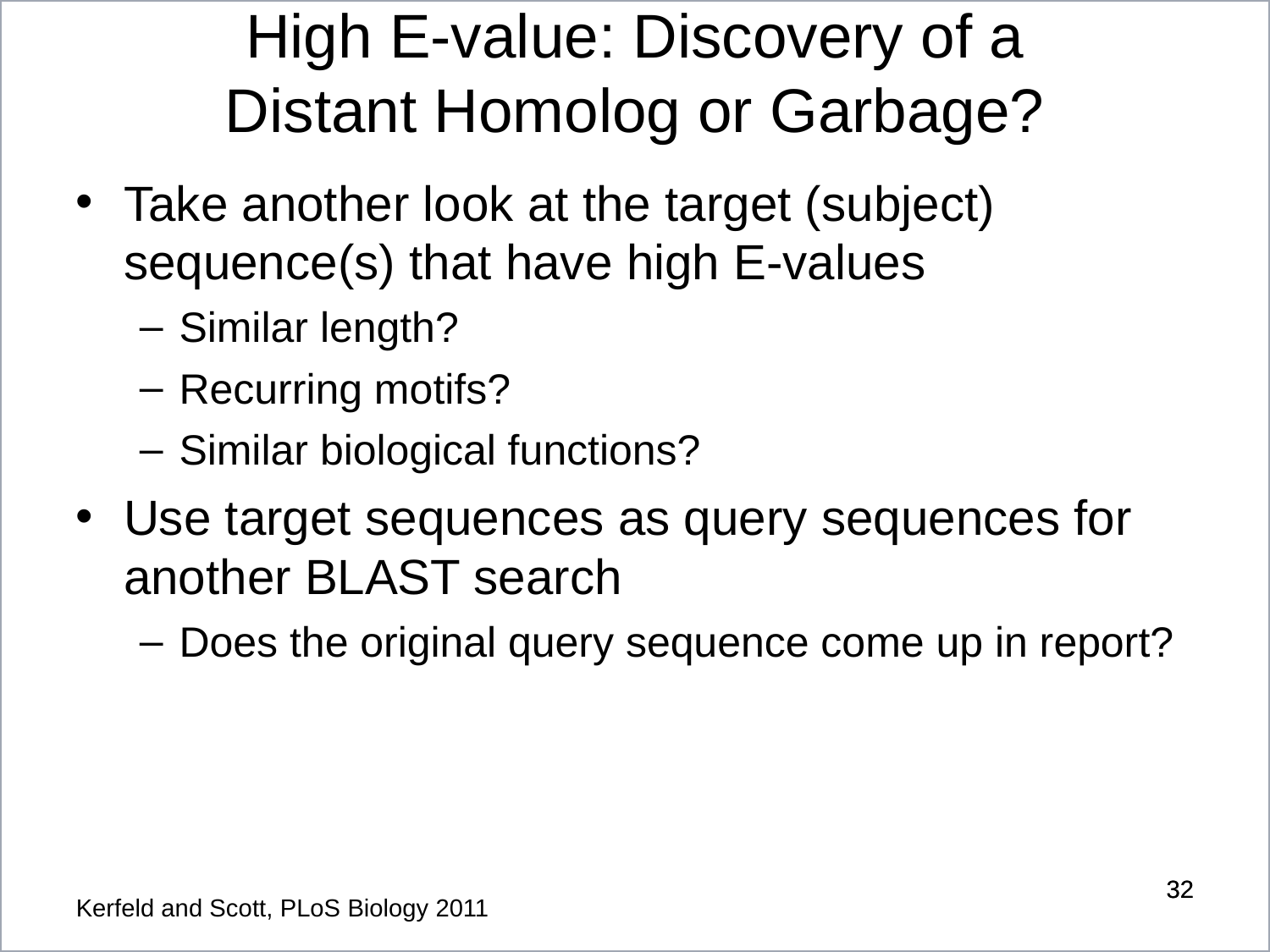

# High E-value: Discovery of a Distant Homolog or Garbage?
Take another look at the target (subject) sequence(s) that have high E-values
Similar length?
Recurring motifs?
Similar biological functions?
Use target sequences as query sequences for another BLAST search
Does the original query sequence come up in report?
<number>
<number>
Kerfeld and Scott, PLoS Biology 2011
