# Supplementary material for: Using BLAST to Teach “E-value-tionary” Concepts
Source: PLoS Biol. 2011 Feb 1;9(2):e1001014. doi: 10.1371/journal.pbio.1001014 (PMC3032543; doi:10.1371/journal.pbio.1001014)
Supplement: Text S1 — Acquiring a feel for the algorithm: manipulating the parameters of BLAST and other experiments. (DOCX) [file pbio.1001014.s002.docx]

**Supplementary Information: Acquiring a feel for the algorithm: manipulating the parameters of BLAST and other experiments**

The exercises rely primarily on BLAST queries of the NCBI nonredundant database (http://www.ncbi.nlm.nih.gov/); however, many public databases are entered via a BLAST interface and so can be used in a similar manner, provided the interface provides a means to tailor the search parameters.

Obtaining a sequence for analysis from the NCBI nonredundant database. BLAST assumes that one has a sequence in hand with which to search a database. To find sequences to use for a BLAST query, students should go to NCBI (http://www.ncbi.nlm.nih.gov/). The search box provided there can be used for keyword searches, in which a student can type keywords of interest. Alternatively, they can type accession numbers into this box and be directed to sequences that might be related to the course content.

Some ‘leads’ are provided here:

Accession number Protein description

NP_440048 potential FMN-protein from a cyanobacterium

This is the example used in figure 1.

2P58_A *Yersinia pestis* type III secretion system needle protein

A necessary component of the pathogenicity factor for the causative agent of

Black Death

P02982 *Escherichia coli* tetracycline resistance protein

Of general interest; also has multiple transmembrane alpha helices which can

be masked for a BLAST search

P02945 Bacteriorhodopsin

Of general interest; typically discussed in introductory microbiology classes.

Has multiple transmembrane alpha helices.

Once a keyword or accession number has been entered into the search box, hits in all the NCBI-accessible databases will be listed. If an accession number was used as a query, next to the icon for the ‘Protein’ database, a ‘1’ will appear. When the student clicks on this ‘1’ link, they will be taken to the web page for this sequence, from which they can obtain the sequence itself, as well as to run a BLAST query of the NCBI databases. If they hit the ‘Run BLAST’ link on this page, they will be taken to a BLAST query page in which the query sequence accession number is already present in the query box. Scrolling down from the query box, students will see a dropdown menu to use to select a database to search. There also is an ‘algorithm parameters’ link which, when selected, allows the student to alter the expect threshold, word size, matrix, gap costs, and whether to use filters and masks.

Exercise 1: BLASTing nucleotide sequences vs. amino acid sequences

To demonstrate that amino acid sequences are more conserved than nucleotide sequences, students can query the nonredundant database at NCBI with both the nucleotide sequence, as well as the translated amino acid sequence, of a gene that is reasonably well-conserved and present across many taxa. Often even the highest-scoring subject sequences retrieved using the nucleotide sequence will cover only small regions of the query sequence, while quite often the corresponding sequences retrieved using the amino acid sequence will cover more of the gene. It is informative for the students to discuss why this is the case. The subject genes from both the nt and aa search could be examined for genes that are retrieved by both searches, and the percent identity of the query sequence versus each subject sequence could be noted.

Exercise 2: Changing the word size

One simple exercise is to have the students conduct a series of BLAST queries using different word sizes, and note the effect of altering this parameter on the nature of the subject sequences retrieved. They should create a table that lists each query by the word size used, number of subject sequences retrieved, and the E-value of the alignment with the poorest-matching subject sequence (e.g., the one with the largest E-value). The BLASTn interface at NCBI is ideal for this exercise, as it allows a range of word sizes (16 – 256 nt).

Exercise 3: Changing the substitution matrix

For this exercise, students will try two different substitution matrices (e.g., BLOSUM 45 vs. BLOSUM 80), and scan the list of subject sequences for those that have been retrieved from both searches. The pairwise alignment between the subject sequence and query sequence includes both the raw score as well as the bit score. Students should note both, and collect them for sequences that share high, moderate, and low identities, to determine which score(s) are most sensitive to matrix selection.

Exercise 4: Manually calculating the raw score for a pairwise alignment

This exercise reinforces a student’s understanding of S values, helps them identify regions in their alignment that had the most impact (for better/worse) on S and provides a connection to the role of specific amino acids side chains in protein function. Students should be provided with a pairwise alignment between two short (~50 aa or nt) sequences; this alignment could be generated from the local alignment tool available at NCBI (bl2seq). Students could use the BLOSUM62 matrix (3) with standard gap costs (11 to open, 1 per residue to extend) to score each position in the alignment and determine the raw score for the alignment. In order to reinforce the relative importance of the gap opening and extension penalties, these may also be varied and the results compared and the resulting alignments inspected. If possible, it would be good to include part of an active site, or otherwise conserved region, to show the relative impacts of well-conserved versus divergent regions on raw scores.

Exercise 5: Comparing the results of a local vs. global alignment

To visualize the difference between a local and global alignment, students could align two sequences using a local alignment tool (e.g., bl2seq at NCBI), and also a global alignment tool (Needleman-Wunsch, also available at NCBI). If the two sequences are cleverly chosen to share only a single domain with common ancestry (e.g., two histidine kinases, or methyl-accepting chemotaxis proteins), the results will be particularly informative for the students, particularly if they are asked to determine which alignment might be more appropriate, and which provides spurious results.

Exercise 6: Changing the E-value threshold

In order to cement the notion that small E-values indicate that the query and subject sequences are very similar to each other, students can undertake a series of BLAST searches in which they change the E-value threshold for the search and record the number of sequences retrieved, as well as the bit score for the poorest alignment. Selecting a small E-value threshold will result in the retrieval only of those sequences with the highest degree of similarity to the query.

Exercise 7: Changing the database

Sometimes students conflate NCBI with BLAST. To help them understand that BLAST is a tool, and can be used to query multiple databases (not just the nonredundant database), students could query multiple databases (e.g., Reference proteins, Swissprot, Protein Data Bank, via NCBI, or completely separate databases, such as Integrated Microbial Genomes). They could compare the number of sequences retrieved with scores above a particular threshold value, which is, if search parameters are constant, a measure of database size.

**Suggested Quiz Questions (for pre- and post exercise administration):**

1. What is the difference between a global and a local alignment?
2. What are three factors that influence the magnitude of the values in a BLOSUM matrix of amino acid substitutions?
3. What would happen to an alignment if gap penalties were infinite? What about if they were set equal to zero?
4. What three factors should you consider in evaluating the quality of an alignment (Evalue, length of alignment)
5. Name one parameter you can vary to optimize your BLAST to identify evolutionarily distant homologs, and describe why changing this parameter would be helpful to find these distant homologs.
6. In your own words, describe what an e-value is.

Is it possible to prove or disprove that two sequences are homologs? What is the best that can be done, and what sort of evidence can be collected?
